# Supplementary material for: Can you refuse these discounts? An evaluation of the use and price discount impact of price-related promotions among US adult smokers by cigarette manufacturers
Source: BMJ Open. 2014 Jun 4;4(6):e004685. doi: 10.1136/bmjopen-2013-004685 (PMC4054636; doi:10.1136/bmjopen-2013-004685)
Supplement: Author's manuscript [file bmjopen-2013-004685.draft_revisions.pdf]

Can you refuse these discounts? An analysis of price-related promotions by U.S. cigarette companies

Journal:	*BMJ Open*
Manuscript ID:	bmjopen-2013-004685
Article Type:	Research
Date Submitted by the Author:	13-Dec-2013
Complete List of Authors:	Caraballo, Ralph; CDC, Office on Smoking and Health<br>Wang, Xu; CDC, Office on Smoking and Health<br>Xu, Xin; CDC, Office on Smoking and Health
**Primary Subject Heading**:	Smoking and tobacco
Secondary Subject Heading:	Health economics, Epidemiology
Keywords:	EPIDEMIOLOGY, HEALTH ECONOMICS, PUBLIC HEALTH

SCHOLARONE™ Manuscripts

For peer review only

For peer review only - http://bmjopen.bmj.com/site/about/guidelines.xhtml

Can you refuse these discounts? An analysis of price-related promotions by U.S. cigarette companies

Ralph S. Caraballo, PhD[1], Xu Wang, PhD[1], Xin Xu, PhD[1]

[1]National Center for Chronic Disease Prevention and Health Promotion, Office on Smoking and Health, Centers for Disease Control and Prevention, Atlanta, GA

Corresponding Author:

Ralph S. Caraballo, PhD
Office on Smoking and Health
National Center for Chronic Disease Prevention and Health Promotion
Centers for Disease Control and Prevention
4770 Buford Highway, NE, MS K-50
Atlanta, Georgia 30341-3717
Phone: 770-488-5723
Fax: 770-488-5767
E-mail: rfc8@cdc.gov

The findings and conclusions in this report are those of the authors and do not necessarily represent the official position of the Centers for Disease Control and Prevention

Targeted journal: **British Medical Journal (BMJ) Open Access**
Abstract: Word count: (journal-recommended maximum of 300 words) is 294
Word count: (journal-recommended maximum of 4,000 words) is 2,819
21 references (25 allowed)
2 tables and 1 figure (5 total allowed)

For peer review only - http://bmjopen.bmj.com/site/about/guidelines.xhtml

ABSTRACT (word limit 294/300):

Objectives: Raising unit price of cigarette has been shown to be one of the most effective ways of reducing cigarette consumption and increase rates of successful quitting. However, researches have shown that price-sensitive smokers have used a variety of strategies to mitigate the effect of rising price of cigarettes on their smoking habits. In particular, 23%-34% of adult smokers in the U.S. use cheaper brands, and 18%-55% use coupons or promotions. Although some studies have assessed smokers' social-demographic characteristics by their preferences to generic brands or premium brands, little is known about the discount use by type of brands. As such, the main purpose of this analysis is to evaluate the uses and effects of these price-related discounts by manufacturer and major brand.

Setting: An analysis based on the cross-sectional 2009-2010 National Adult Tobacco Survey (NATS).

Participants: 11,766 current smokers aged 18 or above in the U.S.

Primary outcome measures: Price-related discount was defined as smokers who used coupons, rebates, buy 1 get 1 free, 2 for 1, or any other special promotions for their last cigarettes purchase.

Results: The use of price-related discounts and associated price impact vary widely by cigarette manufacturer and brand. Approximately 1 of 3 Camel, 1 of 4 Marlboro, and 1 of 8 Newport smokers used price-related discounts in their last cigarette purchases. The average price reductions of discounts offered by Philip Morris (PM) or R.J. Reynolds (RJR) were around 29 cents per pack while that of Lorillard (only Newport brand) was 24 cents per pack. Cigarette brands that provided significant per pack price reductions include: PM Marlboro (28 cents), RJR brands Camel (41 cents), Doral (50 cents), Kool (73 cents), and Salem (80 cents), and Lorillard Newport (24 cents).

For peer review only - http://bmjopen.bmj.com/site/about/guidelines.xhtml

Conclusion: Policies that decrease price-minimization strategies will benefit public health.

Strengths and limitations of this study:

Strengths:

- Research has shown that increasing the unit price of cigarettes is among the most effective public health interventions to reduce cigarette consumption, prevent smoking initiation, and increase rates of successful quitting. This is the first national study in the United States to evaluate the uses and effects of price-related discounts (coupons, rebates, buy 1 get 1 free, 2 for 1, or any other special promotions for the last pack of cigarettes purchased) by U.S. cigarette manufacturer and specific cigarette brand.
- The U.S. national study consisted of an analysis of telephone and cell phone cross sectional data (2009-2010) of 11,766 current cigarette smokers aged 18 or above.
- Price paid for last pack of cigarettes during the past 30 days was collected. Because of recent (last pack bought in past 30 days and most smokers are daily smokers), recall bias should not be a major problem in this study.

Limitations:

- The study design is cross-sectional. Therefore, the study findings may be specific only to the period October 2009–June 2010.
- 4,008 respondents were excluded from the analysis because they failed to provide price paid for their latest purchase, did not report the number of cigarettes smoked per day on smoking days, or failed to report using price minimization strategies.
- The 2009-2010 NATS does not collect information for all price minimization strategies, including cigarette purchases from states with lower price

For peer review only

For peer review only - http://bmjopen.bmj.com/site/about/guidelines.xhtml

Cigarette use is the most preventable cause of death and disease in the United States and presents a significant public health burden.[1] Research has shown that increasing the unit price of cigarettes is among the most effective public health interventions to reduce cigarette consumption, prevent smoking initiation, and increase rates of successful quitting.[2-7] In addition, recent evidence shows that the 2009 federal tobacco excise tax increases have been one of the strategies that have substantially reduced the number of cigarette and smokeless tobacco users among U.S. middle and high school students.[8]

Internal documents from cigarette companies have shown that cigarette companies are aware of the potential impacts that price increases have on their sales and profits. Cigarette companies have developed a variety of price-reduction marketing efforts to promote cigarette sales, such as multipack discounts, rebates, and coupons.[9] According to the most recent cigarette report from the Federal Trade Commission, in addition to giving away 50 million cigarettes for free in 2010, the major cigarette manufactures spent approximately $8.05 billion marketing their products. More than 80% of the marketing expenditures (6.49 billion) went to price-related discounts and promotional allowances used to reduce the retail price of cigarettes.[10]

These cigarette companies' price-related discounts may diminish the public health benefit associated with increased cigarette prices among some smokers even after federal law has raised the unit price of cigarettes. Several recent studies have shown that a large portion of U.S. adult smokers (18%–55%) have taken advantage of these price-related discounts offered by some cigarette companies.[11-16] In addition, evidence from other studies has shown that smokers who used these price-related discounts were less likely to make quit attempts or to successfully quit in the future.[17,18] Although studies have previously investigated demographics and socioeconomic characteristics of smokers who used price minimization strategies, including using coupons or

For peer review only

other types of discounts from cigarette companies,[11-15] little is known about how these price-related discounts affect the average price paid per cigarette when factoring in discounts offered by specific cigarette companies or when looking at specific cigarette brands. Cigarette companies may be directly influencing the prices of their products by using these types of marketing strategies.

Using unique data from the 2009–2010 National Adult Tobacco Survey (NATS) about cigarette brands and price-related discounts used by adult smokers, we evaluated the uses and effects of these price-minimization strategies by cigarette manufacturer and major brand. To the best of our knowledge, this is the first study to provide these estimates from a national representative sample of U.S. adult smokers. The findings of the analysis may help policy and public health stakeholders to further understand the promotion strategies of leading U.S. cigarette companies.

METHODS

Data source

The 2009–2010 NATS is a stratified, national, landline and cell phone survey conducted during October 2009–June 2010. The survey population is a representative sample of non-institutionalized adults aged 18 years or older at both state and national levels. The survey was developed by the Office on Smoking and Health at the Centers for Disease Control and Prevention and was designed primarily to assess the prevalence of tobacco use and the factors related to tobacco use among U.S. adults. The survey has 130 questions that provide information about demographics, health status, cigarette smoking behaviors, price minimization behaviors, cigarette brands preference, the use of other tobacco products, and smoking quit attempts. The

For peer review only - http://bmjopen.bmj.com/site/about/guidelines.xhtml

2009–2010 NATS completed a total of 118,581 interviews, including 110,634 by landline and 7,947 by cell phone. Because samples used for this analysis contain only de-identified observations, this research did not involve human subjects, as defined by Title 45 Code of Federal Regulations, Part 46, and institutional review board (IRB) approval was not required.

This analysis is restricted to current smokers who reported the cigarette brand name that they smoked most often during the past 30 days (n=16,015). Current smokers were defined as those who reported smoking at least 100 cigarettes in their lifetime and currently smoked every day or some days (n=16,542). Among them, respondents who failed to report a brand name were excluded (n=523). Because of the concern of small sample size (n=4), respondents who smoked Forsyth, which is a private brand label, were also excluded from the analysis.

In addition, respondents who failed to provide information on price paid for their latest purchase (n=978), the use of price-minimization strategies (n=2,794), demographic characteristics (age, race/ethnicity, gender, education, marital status, or employment status) (n=477), or time to first cigarette since wake up, were also excluded. The final sample size is 11,766.

Measures of brands and companies

In the survey, respondents were asked about the cigarette brand that they used most often during the past 30 days. A total of 17 brand choices were listed. Except Forsyth and the choice of other brands (n=3,299), the remaining 15 brand names are categorized as premium brands or generic brands. Premium brand names include Camel, Kool, Marlboro, Newport, Pall Mall, Parliament, Salem, Virginia Slims and Winston, and generic brands include Basic, Doral, GPC, Misty, Sonoma, and USA Gold.

For peer review only

For peer review only - http://bmjopen.bmj.com/site/about/guidelines.xhtml

To evaluate price-related discounts and promotions used by major companies, three major cigarette companies were identified on the basis of the 15 brand names above. They are Philip Morris (PM), R.J. Reynolds (RJR), and Lorillard. These companies jointly represented approximately 85% of total U.S. cigarette sales in 2010.[19] PM's brand names include Basic, Marlboro, Parliament, and Virginia Slims. Camel, Doral, GPC, Kool, Misty, Pall Mall, Salem, and Winston are brand manufactured by RJR. Lorillard has the brand Newport. The remaining brands, including Sonoma, USA Gold, and the choice of others brands, are included for other cigarette companies.

Measures of prices and discounts from the industry

The 2009–2010 NATS contains two types of price data. Current smokers who bought cigarettes by packs in their latest purchases were asked to report price paid per pack (after discounts or coupons) in dollars. Those who bought cigarettes by cartons were asked to report price paid per carton. Consequently, price per carton was divided by 10 to obtain a consistent measure of price paid per pack.

In the survey, current smokers were also asked whether they had taken advantage of coupons, rebates, buy-1-get-1-free, 2-for-1, or any other special promotions for cigarettes during the most recent purchase. These coupon and other discount offers were defined in the analysis as coupons and price-related discounts from the industry. Positive responses to this question were used to estimate the prevalence of usage of price-related discounts and promotions by manufacturer and brand name.

For peer review only

Statistical analysis

Cigarette prices reported in the 2009–2010 NATS may reflect the price paid by a smoker after using multiple price minimization strategies. To assess the independent price reduction associated with coupons and other price-related discounts directly from the industry, regression analysis was used to obtain adjusted average prices per pack. The dependent variable in the analysis is the price paid per pack of cigarettes. In addition to the variable of the use of a coupon and other price-related discounts during the most recent purchase, other covariates included the use of premium or generic brands in the past 30 days, purchase of latest cigarette by carton or by pack, purchase on Indian reservations during the previous year, and purchase through the Internet during the previous year.

Daily smoking and time to first cigarette of the day were included in regression analysis as measures of smoking intensity and nicotine dependence so as to control for other price minimization strategies that are not included in the survey, because heavy or more addicted smokers are more likely to use price minimization strategies.[11,12,14,15] Gender, age, race/ethnicity, education, marital and employment status, and state dummy indicators were also included to account for individual differences and policy variations across states. Thus, the constant in the regression analysis presents the adjusted average per pack price before using any price minimization strategies, and the coefficient reflects the price reduction associated with price-related discounts directly from the industry. All analyses were performed using STATA (version 13) and weighted using national NATS weights.

For peer review only - http://bmjopen.bmj.com/site/about/guidelines.xhtml

RESULTS

Overall, among 11,766 adult current smokers, 38.4% identified Marlboro as the brand they used most often (Figure 1), followed by Newport (15.1%) and Camel (8.7%). The percentage of users of other identified brand names were all less than 5%, respectively, ranging from Pall Mall (4.9%) to GPC (0.5%). The combined remaining 15.2% of smokers usually smoked cigarette brands (classified as other brands) that were not identified in the 2009–2010 NATS.

Table 1 presents the use of coupons or other price-related discounts among adult smokers by cigarette manufacturer. Specifically, 43.4% (4,850) reported usually smoking cigarettes produced by PM, 23.9% (3,274) usually smoked cigarettes from RJR, and 15.0% (960) usually smoked Newport cigarettes (Lorillard) (Table 1). The remaining 17.7% (2,682) smoked cigarettes from other companies, including Sonoma and USA Gold, which do not belong to the three major cigarette companies identified in the analysis. Approximately 24.4% of U.S. adult smokers who smoked PM brands used coupons or other price-related discount offers from the company during their most recent cigarette purchase, compared with 21.0% of those who smoked RJR brands, 13.7% of those who smoked Newport (Lorillard), and 11.1% of those who smoked brands from other cigarette companies.

After adjusting for the use of multiple price minimization strategies, respondents' demographic characteristics, smoking intensity, and state policy variations, the average per pack prices paid for cigarettes from PM, RJR, Newport (Lorillard), and other companies were $5.06, $4.63, $4.75, and $3.94, respectively. The average price reductions of coupons or other discounts offered by PM or RJR were statistically significant and similar in magnitude, 29 cents per pack. That of Newport (Lorillard) cigarettes was 24 cents per pack and was marginally significant.

For peer review only - http://bmjopen.bmj.com/site/about/guidelines.xhtml

Table 2 presents the use of coupons or other price-related discounts by specific brand (10 self-reported leading brands only) from the 3 leading cigarette manufacturing companies in the United States. Table 2 is ordered by PM brands first, followed by RJR brands, and last by Newport, the only Lorillard brand listed. The prevalence of using coupons or other price-related discounts varied from 33.9% (Camel, RJR), to 25.6% (Marlboro, PM), 13.7% (Newport, Lorillard), and 10.5% the lowest (Salem, RJR). Thus, about 1 of 3 Camel smokers used these strategies during the last time they bought their cigarettes, compared to about 1 of 4 Marlboro smokers, and about 1 of 8 Newport smokers. The percent price reductions due to use of coupons or discounts ranged from 1.1% (Basic) to 17.3% (Kool). Among these 10 leading U.S. cigarette brands, the average price paid per pack not using any price minimization strategies ranged from paying $3.19 (Pall Mall, RJR) to $6.49 (Virginia Slims, PM). After using coupons or other price-related discounts, the average prices paid for a pack of cigarettes of the top 3 selling brands were 28 cents less for Marlboro ($4.72 instead of $5.00), 41 cents less for Camel ($4.83 instead of $5.24) and 24 cents less for Newport ($4.51 instead of $4.75). Also, those who smoked Salem and used coupons or other price-related discounts saved 80 cents the last time they purchased it. Finally, among these 10 leading U.S. brands, users of Camel (RJR), Marlboro (PM), and Basic (PM) used price minimization strategies the most.

Discussion:

Coupons or other price-related discounts from PM, RJR, and Lorillard were used by 14%-25% of their consumers and have provided price reductions for the smokers who used specific brands. Coupons or other price-related discounts from other companies did not result in statistically significant price per pack reductions for their consumers. To put the range of price reductions associated with coupons or other price-related discounts into context, the cigarette

For peer review only

For peer review only - http://bmjopen.bmj.com/site/about/guidelines.xhtml

federal tax was $1.01 per pack starting April 1st, 2009, and the weighted average state cigarette excise tax rate was $ 1.17 per pack in 2010. These numbers imply that the coupon discounts from the three leading cigarette companies (about 24–29 cents) offset 23.8%–28.7% of public health impacts from the federal tax or 11.0%–13.3% of the impacts from the federal and state excise taxes combined to the smokers of cigarettes produced by these manufactures. As a result, these offers brought actual average prices down for users of specific brands. Therefore, after controlling for the use of other price minimization strategies and respondents' smoking intensity and nicotine addition, the 3 leading cigarette companies provided price reductions for their products through coupons or other price-related discounts.

Although it is true that statistically significant or marginally statistically significant price reductions were observed for the 3 leading U.S. cigarette companies, significant reductions are brand specific. The significant reductions were observed only for Marlboro (U.S. leading brand), Camel (second U.S. leading brand), Kool, Doral and Salem, while marginally for Newport (third U.S. leading brand). Thus, PM, RJR, and Lorillard have concentrated their efforts to provide price discounts mainly to their bestselling brands. This might be one of the reasons that a previous analysis failed to identify significant price reductions associated with promotional offers.[12]

This study has some limitations. First, the study design is cross-sectional. Therefore, the study findings may be specific only to the period October 2009–June 2010. However, the study covers the entire United States, and there is a variety of prices as a function of brand smoked and other factors. Second, most variables in the analysis are collected from recent purchases (i.e., price paid, coupon and other price-related discounts, and carton purchase are for the latest purchase; premium or generic brands are for the last 30 days), but others are collected with a

For peer review only - http://bmjopen.bmj.com/site/about/guidelines.xhtml

time frame of 1 year (i.e., purchase on Indian reservations, purchase through Internet). However, when we excluded Indian reservation and Internet purchases from the analysis, the adjusted average prices and price discounts associated with coupons did not change much. Third, 4,008 respondents were excluded from the analysis because they failed to provide price paid for their latest purchase, did not report the number of cigarettes smoked per day on smoking days, or failed to report using price minimization strategies. Only approximately 20% of respondents interviewed during the first 2 months of the survey were asked if purchases had been made on an Indian reservation anytime during the past year. However, another study has shown that dropping these observations does not significantly affect the results.[16] Fourth, as noted above, the 2009-2010 NATS does not collect information for all price minimization strategies, including cigarette purchases from states with lower price. Although the cross-border purchase is an issue in tobacco control, the prevalence of this behavior was quite low in the United States compared to other forms of price minimization strategies. For example, data from the 2003 and 2006-2007 Tobacco Use Supplement to the Current Population Survey (TUS-CPS) suggests that about 5% of smokers made purchase across a state border,[20] while in the 2010-2011 TUS-CPS, approximately 3.0% of smokers purchased cigarettes from non-tribal land in lower-taxed and non-residential states (estimates not shown). In order to account for unmeasured price minimization strategies, we controlled for smoking intensity and level of nicotine addiction in the analysis since the literature suggests that these are important risk factors of using any price minimization strategies. Finally, the paid prices were determined by using self-reported information from the smoker, which may be subject to recall bias. However, existing evidence indicates that the average of self-reported prices per pack in the 2009-2010 NATS was very consistent with the corresponding 2009 national average price reported in the Tax Burden on

For peer review only - http://bmjopen.bmj.com/site/about/guidelines.xhtml

Tobacco (TBOT).[16] Another benefit of using self-reported price in this analysis is that we are able to control for the corresponding smoking intensity of each smoker, which is not available in market scanned data but closely related to potential use of unmeasured price minimization strategies.

In addition to cigarette companies directly influencing cigarette retail prices by providing coupons or other price-related discounts, cigarette companies may also indirectly affect cigarette prices by offering discounts to retailers and by promoting cigars or pipe tobacco that can be used in roll-your-own cigarettes.[21] Although these indirect influences are critical in tobacco control and certainly warrant additional studies, the NATS survey did not collect such information thus they are not within the scope of this analysis.

Our results show that the three leading cigarette companies in the United States continue to offer price discounts to smokers of their brands, although these promotions appear to be concentrated among their top-selling cigarette brands. As pointed out earlier, smokers who use price-related discounts are less likely to make quit attempts or to successfully quit in the future. The length (duration) of smoking and the amount of cigarettes smoked per day on days the person smoked is strongly associated with a higher likelihood of developing and dying from a smoking-related disease, such as lung cancer, COPD, or heart attacks.[1] Therefore, even though price minimization strategies may increase sales and profits for cigarette companies, these price discounts are likely preventing or delaying some smokers from permanent cessation. Policies that decrease price-minimization strategies will benefit public health.

For peer review only

Contributorship Statement

All 3 authors meet the authorship criteria:

Ralph Caraballo participated in 1)the conception and design of data; 2) drafting the article or revising it critically for important intellectual content; and 3) final approval of the version to be published.

Xu Wang participated in 1) the conception and analysis and interpretation of data; 2) revising it critically for important intellectual content; and 3) final approval of the version to be published.

Xin Xu participated in 1)the conception and design of data; 2) drafting the article or revising it critically for important intellectual content; and 3) final approval of the version to be published.

Competing Interests

None

Data Sharing Statement

The raw and weighted data set are available to the public. A request can be made to the authors

For peer review only

For peer review only - http://bmjopen.bmj.com/site/about/guidelines.xhtml

References

1. United States Department of Health and Human Services. *The Health Consequences of Smoking: A Report of the Surgeon General*. Atlanta, GA: Centers for Disease Control and Prevention, 2004. http://www.cdc.gov/tobacco/data_statistics/sgr/2004/index.htm. (accessed 14 Dec 2012).
2. Chaloupka FJ, Straif K, and Leon ME. Effectiveness of tax and price policies in tobacco control. *Tob Control* 2011;20:235–8.
3. Chaloupka, FJ, Warner, KE. 2000. The economics of smoking. In Culyer,Anthony, and Joseph Newhouse (eds.), *The Handbook of Health Economics*, v.1B, 1539–1627. Elsevier, Amsterdam.
4. Institute of Medicine. *Ending the Tobacco Problem: A Blueprint for the Na*tion. Washington, DC: National Academies Press; 2007.
5. U.S. Department of Health and Human Services. *Reducing Tobacco Use: A Report of the Surgeon General*. Atlanta, Georgia: U.S. Department of Health and Human Services, Centers for Disease Control Prevention; 2000.
6. U.S. Department of Health and Human Services. *Preventing Tobacco Use Among Youth and Young Adults: a Report of the Surgeon General*. Atlanta, GA: US Department of Health and Human Services, Centers for Disease Control and Prevention; 2012.
7. Task Force on Community Preventive Services. Recommendations regarding interventions to reduce tobacco use and exposure to environmental tobacco smoke.*Am J Prev Med* 2001;20(2S):10–5.
8. Huang J, Chaloupka FJ. The impact of the 2009 Federal Tobacco Excise Tax increase on youth tobacco use. Cambridge, MA: National Bureau of Economic Research; 2012. NBER Working Paper no. 18026.
9. Chaloupka FJ, Cummings KM, Morley CP, Horan JK. Tax, price and cigarette smoking: evidence from the tobacco documents and implications for tobacco company marketing strategies. *Tob Control* 2002;11(Suppl I):i62-i72.
10. U.S. Federal Trade Commission. *Cigarette Report for 2009 and 2010*. Washington, DC: Federal Trade Commission; 2012. http://ftc.gov/os/2012/09/120921cigarettereport.pdf (accessed 4 Feb 2013).
11. Hyland A, Bauer JE, Li Q, Abrams SM, Higbee C, Peppone L, *et al*. Higher cigarette prices influence cigarette purchase patterns. *Tob Control* 2005;14:86–92.
12. White VM, Gilpin EA, White MM, Pierce JP. How do smokers control their cigarette expenditures? *Nicotine Tob Res* 2005;7:625–35.
13. White VM, White MM, Freeman K, Gilpin EA, Pierce JP. Cigarette promotional offers: who takes advantage? *Am J Pre Med* 2006;30:225–31.
14. Choi K, Hennrikus D, Forster J, St. Clair AW. Use of price-minimizing strategies by smokers and their effects on subsequent smoking behaviors. *Nicotine Tob Res* 2012;14(7):864–70.
15. Licht AS, Hyland AJ, O'Connor RJ, Chaloupka FJ, Borland R, Fong GT, *et al*. Socio-economic variation in price minimizing behaviors: findings from the International Tobacco Control (ITC) Four Country Survey. *Int J Environ Res Public Health* 2011;8:234–52.
16. Xu X, Pesko MF, Tynan MA, Gerzoff R, Malarcher AM, Pechacek TF. Cigarette price-minimizing strategies by U.S. smokers. *Am J Pre Med* 2013;30:225–31.
17. Hyland A, Laux FL, Higbee C, Hastings G, Ross H, *et al*. Cigarette purchase patterns in four countries and the relationship with cessation: findings from the International Tobacco Control (ITC) Four Country Survey. *Tob Res* 2006;15(Suppl III):iii59–iii64.
18. Licht AS, Hyland AJ, O'Connor RJ, Chaloupka FJ, Borland R, *et al*. How do price minimizing behaviors impact smoking cessation? findings from the International Tobacco Control (ITC) Four Country Survey. *Int J Environ Res Public Health* 2011;8(5):1671–91.

For peer review only

19. Maxwell JC. *The Maxwell Report: Year End & Fourth Quarter 2010 Sales Estimates for the Cigarette Industry*. Richmond (VA): John C. Maxwell, Jr., 2011 (accessed 24 Apr 2012).

20. DeCicca P, Kenkel DS, Liu F. Excise tax avoidance: the case of state cigarette taxes. *J Health Econ* 2013; 32: 1130-1141.

21. Centers for Disease Control and Prevention, Consumption of cigarettes and combustible tobacco-United States, 2000–2011, *Morb Mortal Wkly Rep,* 2012; 61,30, 565–569.

For peer review only

For peer review only - http://bmjopen.bmj.com/site/about/guidelines.xhtml

Figure 1. Brand preference among U.S. adult smokers (2009-2010 NATS)

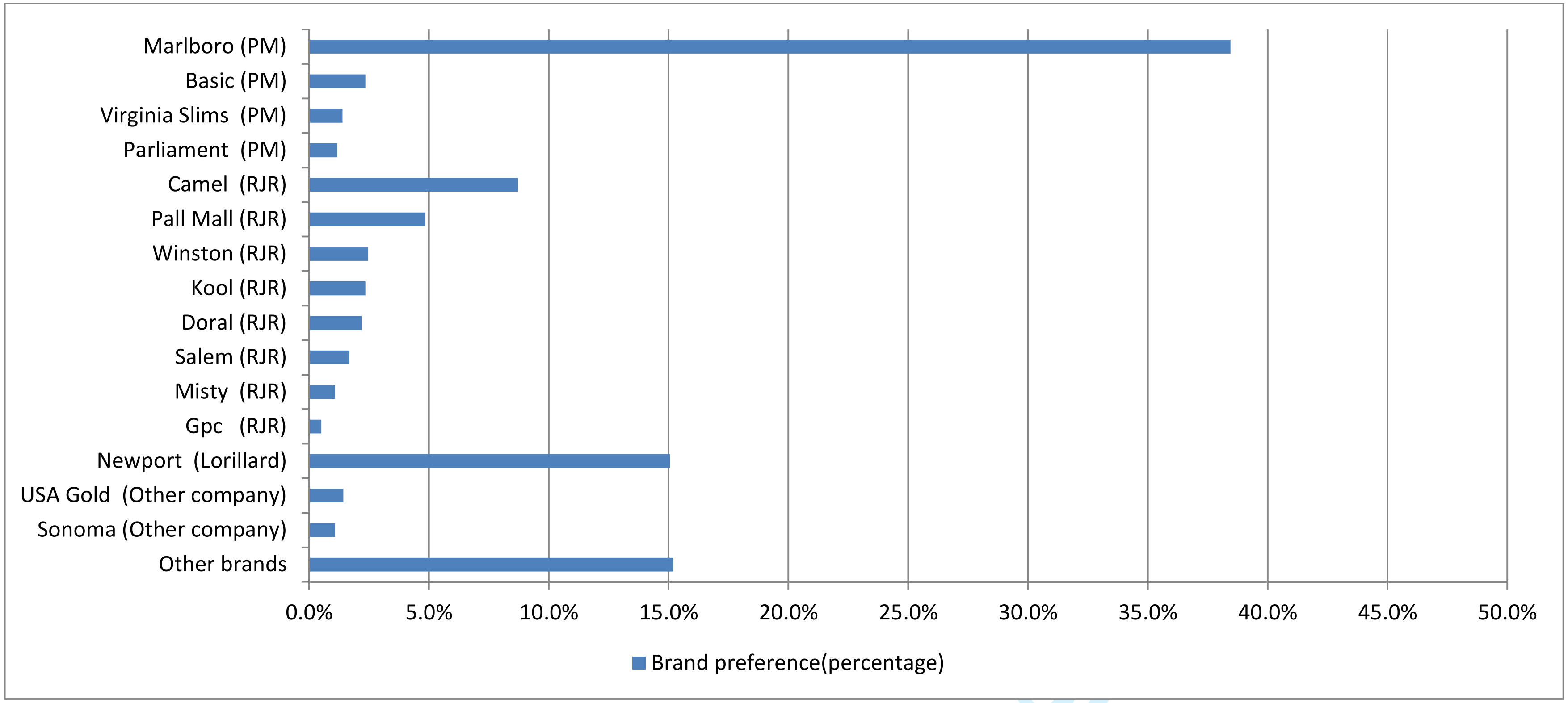

For peer review only - http://bmjopen.bmj.com/site/about/guidelines.xhtml

Table 1. The use of coupons or other price-related discounts by major cigarette manufactures

	PM	RJR	LORILLARD (Newport only)	Other Companies[a]
Prevalence of brand use	43.4%	23.9%	15.0%	17.7%
Prevalence of coupons or discounts used by smokers of that manufacture	24.4%	21.0%	13.7%	11.1%
Price reduction per pack for smokers of that manufacture($)	-0.29**	-0.29**	-0.24*	-0.23
Average price per pack for smokers of that manufacture ($)	5.06	4.63	4.75	3.94
Percentage of discount rendered to smokers of that manufacture	5.7%	6.3%	5.1%	5.8%
N	4,850	3,274	960	2,682

Notes: N represents un-weighted sample size. All estimates were obtained with national weights. In regressions, the dependent variable is price paid per pack, and the variable of interest is whether using coupons, rebates, buy-1-get-1-free, 2-for-1, or any other special promotions for cigarettes in the most recent purchase. Control variables include demographic characteristics (race, age, gender, education, marital status, and employment status), state dummy variables, whether the respondent is a daily smoker, time to first cigarette since wake up, and all other price-minimization behaviors (i.e., purchase of generic brands, purchase of cartons, purchase on Indian reservation, or purchase through the Internet). Percentage of discount rendered is obtained by dividing average price per pack with price reduction associated with coupons and other price-related discounts.

[a] Users of Sonoma, USA Gold brands, and other brand names which not listed in the survey.

**Statistically significant at 5% level; * statistically significant at 10% level.

For peer review only

Table 2. The use of coupons or other price-related discounts by cigarette manufacturer and by top 10 leading U.S. brands

		Prevalence of coupon use	% of discount	Average price	Price after discount	Rank[a]
PM	Basic	22.2%	1.1%	$4.41	$4.36	3
	Marlboro	25.6%	5.6%	$5.00	$4.72**	2
	Virginia Slims	10.6%	3.4%	$6.49	$6.27	9
RJR	Camel	33.9%	7.8%	$5.24	$4.83**	1
	Doral	15.6%	12.7%	$3.93	$3.43**	6
	Kool	11.6%	17.3%	$4.23	$3.50**	8
	Pall Mall	15.9%	1.3%	$3.19	$3.15	5
	Salem	10.5%	15.7%	$5.11	$4.31**	10
	Winston	17.1%	3.7%	$4.07	$3.92	4
Lorillard	Newport	13.7%	5.1%	$4.75	$4.51*	7

[a] Based on prevalence of coupon use.

**Price reduction associated with coupons and other price-related discounts is statistically significant at 5% level, * statistically significant at 10% level.

For peer review only - http://bmjopen.bmj.com/site/about/guidelines.xhtml

BMJ Open

Can you refuse these discounts? An analysis of price-related promotions by U.S. cigarette companies

Journal:	*BMJ Open*
Manuscript ID:	bmjopen-2013-004685.R1
Article Type:	Research
Date Submitted by the Author:	04-Apr-2014
Complete List of Authors:	Caraballo, Ralph; CDC, Office on Smoking and Health<br>Wang, Xu; CDC, Office on Smoking and Health<br>Xu, Xin; CDC, Office on Smoking and Health
**Primary Subject Heading**:	Smoking and tobacco
Secondary Subject Heading:	Health economics, Epidemiology
Keywords:	EPIDEMIOLOGY, HEALTH ECONOMICS, PUBLIC HEALTH

SCHOLARONE™
Manuscripts

For peer review only

Can you refuse these discounts? An analysis of price-related promotions by U.S. cigarette companies

Ralph S. Caraballo, PhD[1], Xu Wang, PhD[1], Xin Xu, PhD[1]

[1]National Center for Chronic Disease Prevention and Health Promotion, Office on Smoking and Health, Centers for Disease Control and Prevention, Atlanta, GA

Corresponding Author:

Ralph S. Caraballo, PhD
Office on Smoking and Health
National Center for Chronic Disease Prevention and Health Promotion
Centers for Disease Control and Prevention
4770 Buford Highway, NE, MS K-50
Atlanta, Georgia 30341-3717
Phone: 770-488-5723
Fax: 770-488-5767
E-mail: rfc8@cdc.gov

The findings and conclusions in this report are those of the authors and do not necessarily represent the official position of the Centers for Disease Control and Prevention

Targeted journal: **British Medical Journal (BMJ) Open Access**
Abstract: Word count: (journal-recommended maximum of 300 words) is 296
Word count: (journal-recommended maximum of 4,000 words) is 3,035
25 references (25 allowed)
2 tables and 1 figure (5 total allowed)

For peer review only
For peer review only - http://bmjopen.bmj.com/site/about/guidelines.xhtml

ABSTRACT (word limit 300/300):

Objectives: Raising unit price of cigarette has been shown to be one of the most effective ways of reducing cigarette consumption and increase rates of successful quitting. However, researches have shown that price-sensitive smokers have used a variety of strategies to mitigate the effect of rising price of cigarettes on their smoking habits. In particular, 23%-34% of adult smokers in the U.S. use cheaper brands, and 18%-55% use coupons or promotions. Although some studies have assessed smokers' social-demographic characteristics by their preferences to generic brands or premium brands, little is known about the discount use by type of brands. As such, the main purpose of this analysis is to evaluate the uses and price discount effects of these price-related discounts by manufacturer and major brand.

Setting: An analysis based on the cross-sectional 2009-2010 National Adult Tobacco Survey (NATS).

Participants: 11,766 current smokers aged 18 or above in the U.S.

Primary outcome measures: Price-related discount was defined as smokers who used coupons, rebates, buy 1 get 1 free, 2 for 1, or any other special promotions for their last cigarettes purchase.

Results: The use of price-related discounts and associated price impact vary widely by cigarette manufacturer and brand. Approximately 1 of 3 Camel, 1 of 4 Marlboro, and 1 of 8 Newport smokers used price-related discounts in their latest cigarette purchases. The average price reductions of discounts offered by Philip Morris (PM) or R.J. Reynolds (RJR) were around 29 cents per pack while that of Lorillard (Newport only) was 24 cents per pack. Cigarette brands that provided significant per pack price reductions include: PM Marlboro (28 cents), RJR brands Camel (41 cents), Doral (50 cents), Kool (73 cents), and Salem (80 cents), and Lorillard Newport (24 cents).

For peer review only

For peer review only - http://bmjopen.bmj.com/site/about/guidelines.xhtml

Conclusion: Policies that decrease price-minimization strategies will benefit public health.

Strengths and limitations of this study:

Strengths:

- Research has shown that increasing the unit price of cigarettes is among the most effective public health interventions to reduce cigarette consumption, prevent smoking initiation, and increase rates of successful quitting. This is the first national study in the United States to evaluate the uses and effects of price-related discounts (coupons, rebates, buy 1 get 1 free, 2 for 1, or any other special promotions for the last pack of cigarettes purchased) by U.S. cigarette manufacturer and specific cigarette brand.
- The U.S. national study consisted of an analysis of telephone and cell phone cross sectional data (2009-2010) of 11,766 current cigarette smokers aged 18 or above.
- Price paid for last pack of cigarettes during the past 30 days was collected. Because of recent (last pack bought in past 30 days and most smokers are daily smokers), recall bias should not be a major problem in this study.

Limitations:

- The study design is cross-sectional. Therefore, the study findings may be specific only to the period October 2009–June 2010.
- 4,008 respondents were excluded from the analysis because they failed to provide price paid for their latest purchase, did not report the number of cigarettes smoked per day on smoking days, or failed to report using price minimization strategies.
- The 2009-2010 NATS does not collect information for all price minimization strategies, including cigarette purchases from states with lower price

For peer review only

For peer review only - http://bmjopen.bmj.com/site/about/guidelines.xhtml

Cigarette use is the most preventable cause of death and disease in the United States and presents a significant public health burden.[1] Research has shown that increasing the unit price of cigarettes is among the most effective public health interventions to reduce cigarette consumption, prevent smoking initiation, and increase rates of successful quitting.[2-7] In addition, recent evidence shows that the 2009 federal tobacco excise tax increases have been one of the strategies that have substantially reduced the number of cigarette and smokeless tobacco users among U.S. middle and high school students.[8]

Internal documents from cigarette companies have shown that cigarette companies are aware of the potential impacts that price increases have on their sales and profits. Cigarette companies have developed a variety of price-reduction marketing efforts to promote cigarette sales, such as multipack discounts, rebates, and coupons.[9] According to the most recent cigarette report from the Federal Trade Commission, in addition to giving away 50 million cigarettes for free in 2010, the major cigarette manufactures spent approximately $8.05 billion marketing their products. More than 80% of the marketing expenditures (6.49 billion) went to price-related discounts and promotional allowances used to reduce the retail price of cigarettes.[10]

These cigarette companies' price-related discounts may diminish the public health benefit associated with increased cigarette prices among some smokers even after federal law has raised the unit price of cigarettes. Several recent studies have shown that a large portion of U.S. adult smokers (18%–55%) have taken advantage of these price-related discounts offered by some cigarette companies.[11-16] In addition, evidence from other studies has shown that smokers who used these price-related discounts were less likely to make quit attempts or to successfully quit in the future.[17-20] Although studies have previously investigated demographics and socioeconomic characteristics of smokers who used price minimization strategies, including using coupons or

For peer review only - http://bmjopen.bmj.com/site/about/guidelines.xhtml

other types of discounts from cigarette companies,[11-15] little is known about how these price-related discounts affect the average price paid per cigarette when factoring in discounts offered by specific cigarette companies or when looking at specific cigarette brands. Cigarette companies may be directly influencing the prices of their products by using these types of marketing strategies.

Using unique data from the 2009–2010 National Adult Tobacco Survey (NATS) about cigarette brands and price-related discounts used by adult smokers, we evaluated the uses and price discount effects of these price-minimization strategies by cigarette manufacturer and major brand. To the best of our knowledge, this is the first study to provide these estimates from a national representative sample of U.S. adult smokers. The findings of the analysis may help policy and public health stakeholders to further understand the promotion strategies of leading U.S. cigarette companies.

METHODS

Data source

The 2009–2010 NATS is a stratified, national, landline and cell phone survey conducted during October 2009–June 2010. The survey population is a representative sample of non-institutionalized adults aged 18 years or older at both state and national levels. The survey was developed by the Office on Smoking and Health at the Centers for Disease Control and Prevention and was designed primarily to assess the prevalence of tobacco use and the factors related to tobacco use among U.S. adults. The survey has 130 questions that provide information about demographics, health status, cigarette smoking behaviors, price minimization behaviors, cigarette brands preference, the use of other tobacco products, and smoking quit attempts. The

For peer review only

2009–2010 NATS completed a total of 118,581 interviews, including 110,634 by landline and 7,947 by cell phone. Because samples used for this analysis contain only de-identified observations, this research did not involve human subjects, as defined by Title 45 Code of Federal Regulations, Part 46, and institutional review board (IRB) approval was not required.

This analysis is restricted to current smokers who reported the cigarette brand name that they smoked most often during the past 30 days (n=16,015). Current smokers were defined as those who reported smoking at least 100 cigarettes in their lifetime and currently smoked every day or some days (n=16,542). Among them, respondents who failed to report a brand name were excluded (n=523). Because of the concern of small sample size (n=4), respondents who smoked Forsyth, which is a private brand label, were also excluded from the analysis.

In addition, respondents who failed to provide information on price paid for their latest purchase (n=978), the use of price-minimization strategies (n=2,794), demographic characteristics (age, race/ethnicity, gender, education, marital status, or employment status) (n=477), or time to first cigarette since wake up, were also excluded. The final sample size is 11,766.

Measures of brands and companies

In the survey, respondents were asked about the cigarette brand that they used most often during the past 30 days. A total of 17 brand choices were listed. Except Forsyth and the choice of other brands (n=3,299), the remaining 15 brand names are categorized as premium brands or generic brands. Premium brand names include Camel, Kool, Marlboro, Newport, Pall Mall, Parliament, Salem, Virginia Slims and Winston, and generic brands include Basic, Doral, GPC, Misty, Sonoma, and USA Gold.

For peer review only - http://bmjopen.bmj.com/site/about/guidelines.xhtml

To evaluate price-related discounts and promotions used by major companies, three major cigarette companies were identified on the basis of the 15 brand names above. They are Philip Morris (PM), R.J. Reynolds (RJR), and Lorillard. These companies jointly represented approximately 85% of total U.S. cigarette sales in 2010.[21] PM's brand names include Basic, Marlboro, Parliament, and Virginia Slims. Camel, Doral, GPC, Kool, Misty, Pall Mall, Salem, and Winston are brand manufactured by RJR. Lorillard has the brand Newport. The remaining brands, including Sonoma, USA Gold, and the choice of others brands, are included for other cigarette companies.

Measures of prices and discounts from the industry

The 2009–2010 NATS contains two types of price data. Current smokers who bought cigarettes by packs in their latest purchases were asked to report price paid per pack (after discounts or coupons) in dollars. Those who bought cigarettes by cartons were asked to report price paid per carton. Consequently, price per carton was divided by 10 to obtain a consistent measure of price paid per pack.

In the survey, current smokers were also asked whether they had taken advantage of coupons, rebates, buy-1-get-1-free, 2-for-1, or any other special promotions for cigarettes during the most recent purchase. These coupon and other discount offers were defined in the analysis as coupons and price-related discounts from the industry. Positive responses to this question were used to estimate the prevalence of usage of price-related discounts and promotions by manufacturer and brand name.

For peer review only - http://bmjopen.bmj.com/site/about/guidelines.xhtml

Statistical analysis

Cigarette prices reported in the 2009–2010 NATS may reflect the price paid by a smoker after using multiple price minimization strategies. To assess the independent price reduction associated with coupons and other price-related discounts directly from the industry, for each cigarette company or brand, regression analysis with the following specification was used to obtain adjusted average prices per pack.

For peer review only

$$Per\ pack\ price\ paid = \beta_1 + \beta_2 discounts + \beta_3 OtherPMS + \beta_4 Ciguse + \beta_5 Demographics + \beta_6 state$$

The dependent variable was per pack price paid for cigarettes. The key independent variable was the dichotomous indicator of using a coupon and other price-related discounts during the most recent purchase (discounts). The covariates of other price minimization strategies (OtherPMS) included the use of premium or generic brands in the past 30 days, purchase of latest cigarette by carton or by pack, purchase on Indian reservations during the previous year, and purchase through the Internet during the previous year. Daily smoking and time to first cigarette of the day (ciguse) were included in regression analysis as measures of smoking intensity and nicotine dependence so as to control for other price minimization strategies that are not included in the survey, because heavy or more addicted smokers are more likely to use price minimization strategies.[11,12,14,15] Gender, age, race/ethnicity, education, marital and employment status (demographics), and state dummy indicators (state) were also included to account for individual differences and policy variations across states.

Thus, the constant, β_1, presents the adjusted average per pack price before using any price minimization strategies, and the coefficient, β_2 reflects the price reduction associated with price-related discounts directly from the industry. All analyses were performed using STATA

For peer review only - http://bmjopen.bmj.com/site/about/guidelines.xhtml

(version 13). Post-stratification sampling weights were incorporated in all analyses to account for the complex survey design of the 2009-2010 NATS and nonresponse.

RESULTS

Overall, among 11,766 adult current smokers, 38.4% identified Marlboro as the brand they used most often (Figure 1), followed by Newport (15.1%) and Camel (8.7%). The percentage of users of other identified brand names were all less than 5%, respectively, ranging from Pall Mall (4.9%) to GPC (0.5%). The combined remaining 15.2% of smokers usually smoked cigarette brands (classified as other brands) that were not identified in the 2009–2010 NATS.

Table 1 presents the use of coupons or other price-related discounts among adult smokers by cigarette manufacturer. Specifically, 43.4% (4,850) reported usually smoking cigarettes produced by PM, 23.9% (3,274) usually smoked cigarettes from RJR, and 15.0% (960) usually smoked Newport cigarettes (Lorillard) (Table 1). The remaining 17.7% (2,682) smoked cigarettes from other companies, including Sonoma and USA Gold, which do not belong to the three major cigarette companies identified in the analysis. Approximately 24.4% of U.S. adult smokers who smoked PM brands used coupons or other price-related discount offers from the company during their most recent cigarette purchase, compared with 21.0% of those who smoked RJR brands, 13.7% of those who smoked Newport (Lorillard), and 11.1% of those who smoked brands from other cigarette companies.

After adjusting for the use of multiple price minimization strategies, respondents' demographic characteristics, smoking intensity, and state policy variations, the average per pack

For peer review only - http://bmjopen.bmj.com/site/about/guidelines.xhtml

prices paid for cigarettes from PM, RJR, Newport (Lorillard), and other companies were $5.06, $4.63, $4.75, and $3.94, respectively. The average price reductions of coupons or other discounts offered by PM or RJR were statistically significant and similar in magnitude, 29 cents per pack. That of Newport (Lorillard) cigarettes was 24 cents per pack and was marginally significant.

Table 2 presents the use of coupons or other price-related discounts by specific brand (10 self-reported leading brands only) from the 3 leading cigarette manufacturing companies in the United States. Table 2 is ordered by PM brands first, followed by RJR brands, and last by Newport, the only Lorillard brand listed. The prevalence of using coupons or other price-related discounts varied from 33.9% (Camel, RJR), to 25.6% (Marlboro, PM), 13.7% (Newport, Lorillard), and 10.5% the lowest (Salem, RJR). Thus, about 1 of 3 Camel smokers used these strategies during the last time they bought their cigarettes, compared to about 1 of 4 Marlboro smokers, and about 1 of 8 Newport smokers. The percent price reductions due to use of coupons or discounts ranged from 1.1% (Basic) to 17.3% (Kool). Among these 10 leading U.S. cigarette brands, the average price paid per pack not using any price minimization strategies ranged from paying $3.19 (Pall Mall, RJR) to $6.49 (Virginia Slims, PM). After using coupons or other price-related discounts, the average prices paid for a pack of cigarettes of the top 3 selling brands were 28 cents less for Marlboro ($4.72 instead of $5.00), 41 cents less for Camel ($4.83 instead of $5.24) and 24 cents less for Newport ($4.51 instead of $4.75). Also, those who smoked Salem and used coupons or other price-related discounts saved 80 cents the last time they purchased it. Finally, among these 10 leading U.S. brands, users of Camel (RJR), Marlboro (PM), and Basic (PM) used price minimization strategies the most.

For peer review only - http://bmjopen.bmj.com/site/about/guidelines.xhtml

Discussion:

Coupons or other price-related discounts from PM, RJR, and Lorillard were used by 14%-25% of their consumers and have provided price reductions for the smokers who used specific brands. Coupons or other price-related discounts from other companies did not result in statistically significant price per pack reductions for their consumers. Therefore, after controlling for the use of other price minimization strategies and respondents' smoking intensity and nicotine addition, the 3 leading cigarette companies provided price reductions for their products through coupons or other price-related discounts.

To put the range of price reductions associated with coupons or other price-related discounts into context, the cigarette federal tax was $1.01 per pack starting April 1st, 2009, and the weighted average state cigarette excise tax rate was $ 1.17 per pack in 2010. These numbers imply that the coupon discounts from the three leading cigarette companies (about 24–29 cents) offset 23.8%–28.7% of the price impacts from the federal tax or 11.0%–13.3% of the price impacts from the federal and state excise taxes combined to the smokers of cigarettes produced by these manufactures. As a result, these offers brought actual average prices down for users of specific brands, while the price reductions associated with these discounts were likely to increase the sales in these companies. For example, with a price elasticity of overall demand for cigarettes among adults at somewhere between -0.3 and -0.7,[22] ceteris paribus, these price discounts can be translated into 10.2-23.9 million packs of cigarette sales in 2010 for Marlboro, 3.0-6.6 million packs for Newport, and 1.8-4.1 million packs for Camel.[21]

Although it is true that statistically significant or marginally statistically significant price reductions were observed for the 3 leading U.S. cigarette companies, significant reductions are brand specific. The significant reductions were observed only for Marlboro (U.S. leading brand), Camel (second U.S. leading brand), Kool, Doral and Salem, while marginally for Newport (third

For peer review only - http://bmjopen.bmj.com/site/about/guidelines.xhtml

U.S. leading brand). Thus, PM, RJR, and Lorillard have concentrated their efforts to provide price discounts mainly to their bestselling brands. This might be one of the reasons that a previous analysis failed to identify significant price reductions associated with promotional offers.[12]

This study has some limitations. First, the study design is cross-sectional. Therefore, the study findings may be specific only to the period October 2009–June 2010. However, the study covers the entire United States, and there is a variety of prices as a function of brand smoked and other factors. Second, most variables in the analysis are collected from recent purchases (i.e., price paid, coupon and other price-related discounts, and carton purchase are for the latest purchase; premium or generic brands are for the last 30 days), but others are collected with a time frame of 1 year (i.e., purchase on Indian reservations, purchase through Internet). However, when we excluded Indian reservation and Internet purchases from the analysis, the adjusted average prices and price discounts associated with coupons did not change much. Third, because of an approval delay, only approximately 20% of respondents interviewed during the first 2 months of the survey were asked if purchases had been made on an Indian reservation anytime during the past year. In subsequent months, this question was asked of more than 90% of respondents. In the full sample, total missing responses for this question were 18.4% (3,503). However, sensitivity analysis has shown that dropping these observations does not significantly affect the results.[16] As noted in the method section, we also excluded respondents who failed to report price paid for their latest purchase, the brand name they used most often in the last 30 days, or some of their demographic characteristics. We compared smoking and social-demographic characteristics between individuals with incomplete information and individuals with complete information and have found little differences at the mean level. Fourth, as noted

For peer review only - http://bmjopen.bmj.com/site/about/guidelines.xhtml

above, the 2009-2010 NATS does not collect information for all price minimization strategies, including cigarette purchases from states with lower price. Although the cross-border purchase is an issue in tobacco control, the prevalence of this behavior was quite low in the United States compared to other forms of price minimization strategies. For example, data from the 2003 and 2006-2007 Tobacco Use Supplement to the Current Population Survey (TUS-CPS) suggests that about 5% of smokers made purchase across a state border,[23] while in the 2010-2011 TUS-CPS, approximately 3.0% of smokers purchased cigarettes from non-tribal land in lower-taxed and non-residential states (estimates not shown). In order to account for unmeasured price minimization strategies, we controlled for smoking intensity and level of nicotine addiction in the analysis since the literature suggests that these are important risk factors of using any price minimization strategies. Finally, the paid prices were determined by using self-reported information from the smoker, which may be subject to recall bias. However, existing evidence indicates that the average of self-reported prices per pack in the 2009-2010 NATS was very consistent with the corresponding 2009 national average price reported in the Tax Burden on Tobacco (TBOT).[16] Another benefit of using self-reported price in this analysis is that we are able to control for the corresponding smoking intensity of each smoker, which is not available in market scanned data but closely related to potential use of unmeasured price minimization strategies.

In addition to cigarette companies directly influencing cigarette retail prices by providing coupons or other price-related discounts, cigarette companies may also indirectly affect cigarette prices by offering discounts to retailers and by promoting cigars or pipe tobacco that can be used in roll-your-own cigarettes.[24] Although these indirect influences are critical in tobacco control

For peer review only - http://bmjopen.bmj.com/site/about/guidelines.xhtml

and certainly warrant additional studies, the NATS survey did not collect such information thus they are not within the scope of this analysis.

Our results show that the three leading cigarette companies in the United States continue to offer price discounts to smokers of their brands, although these promotions appear to be concentrated among their top-selling cigarette brands. Cigarette companies can be strategic when offering price discounts. For example, existing literature suggests that young adults, females, and heavy smokers are more frequently targeted for these promotions.[19] Other study have shown that cigarette brands with low market share target young adults with the goal of encouraging brand switching, while major brands target older smokers to facilitate brand loyalty.[25] As pointed out earlier, smokers who use price-related discounts are less likely to make quit attempts or to successfully quit in the future. The length (duration) of smoking and the amount of cigarettes smoked per day on days the person smoked is strongly associated with a higher likelihood of developing and dying from a smoking-related disease, such as lung cancer, COPD, or heart attacks.[1] Therefore, even though price minimization strategies may increase sales and profits for cigarette companies, these price discounts are likely preventing or delaying some smokers from permanent cessation. Policies that decrease price-minimization strategies will benefit public health.

For peer review only - http://bmjopen.bmj.com/site/about/guidelines.xhtml

Contributorship Statement

All 3 authors meet the authorship criteria:

Ralph Caraballo participated in 1)the conception and design of data; 2) drafting the article or revising it critically for important intellectual content; and 3) final approval of the version to be published.

Xu Wang participated in 1) the conception and analysis and interpretation of data; 2) revising it critically for important intellectual content; and 3) final approval of the version to be published.

Xin Xu participated in 1)the conception and design of data; 2) drafting the article or revising it critically for important intellectual content; and 3) final approval of the version to be published.

Competing Interests

None

Data Sharing Statement

The raw and weighted data set are available to the public. A request can be made to the authors

For peer review only

For peer review only - http://bmjopen.bmj.com/site/about/guidelines.xhtml

References

1. United States Department of Health and Human Services. *The Health Consequences of Smoking: A Report of the Surgeon General*. Atlanta, GA: Centers for Disease Control and Prevention, 2004. http://www.cdc.gov/tobacco/data_statistics/sgr/2004/index.htm. (accessed 14 Dec 2012).
2. Chaloupka FJ, Straif K, and Leon ME. Effectiveness of tax and price policies in tobacco control. *Tob Control* 2011;20:235–8.
3. Chaloupka, FJ, Warner, KE. 2000. The economics of smoking. In Culyer,Anthony, and Joseph Newhouse (eds.), *The Handbook of Health Economics*, v.1B, 1539–1627. Elsevier, Amsterdam.
4. Institute of Medicine. *Ending the Tobacco Problem: A Blueprint for the Na*tion. Washington, DC: National Academies Press; 2007.
5. U.S. Department of Health and Human Services. *Reducing Tobacco Use: A Report of the Surgeon General*. Atlanta, Georgia: U.S. Department of Health and Human Services, Centers for Disease Control Prevention; 2000.
6. U.S. Department of Health and Human Services. *Preventing Tobacco Use Among Youth and Young Adults: a Report of the Surgeon General*. Atlanta, GA: US Department of Health and Human Services, Centers for Disease Control and Prevention; 2012.
7. Task Force on Community Preventive Services. Recommendations regarding interventions to reduce tobacco use and exposure to environmental tobacco smoke.*Am J Prev Med* 2001;20(2S):10–5.
8. Huang J, Chaloupka FJ. The impact of the 2009 Federal Tobacco Excise Tax increase on youth tobacco use. Cambridge, MA: National Bureau of Economic Research; 2012. NBER Working Paper no. 18026.
9. Chaloupka FJ, Cummings KM, Morley CP, et al. Tax, price and cigarette smoking: evidence from the tobacco documents and implications for tobacco company marketing strategies. *Tob Control* 2002;11(Suppl I):i62-i72.
10. U.S. Federal Trade Commission. *Cigarette Report for 2009 and 2010*. Washington, DC: Federal Trade Commission; 2012. http://ftc.gov/os/2012/09/120921cigarettereport.pdf (accessed 4 Feb 2013).
11. Hyland A, Bauer JE, Li Q, Abrams SM, Higbee C, Peppone L, *et al*. Higher cigarette prices influence cigarette purchase patterns. *Tob Control* 2005;14:86–92.
12. White VM, Gilpin EA, White MM, et al. How do smokers control their cigarette expenditures? *Nicotine Tob Res* 2005;7:625–35.
13. White VM, White MM, Freeman K, et al. Cigarette promotional offers: who takes advantage? *Am J Pre Med* 2006;30:225–31.
14. Choi K, Hennrikus D, Forster J, et al. Use of price-minimizing strategies by smokers and their effects on subsequent smoking behaviors. *Nicotine Tob Res* 2012;14(7):864–70.
15. Licht AS, Hyland AJ, O'Connor RJ, et al. Socio-economic variation in price minimizing behaviors: findings from the International Tobacco Control (ITC) Four Country Survey. *Int J Environ Res Public Health* 2011;8:234–52.
16. Xu X, Pesko MF, Tynan MA, et al. Cigarette price-minimizing strategies by U.S. smokers. *Am J Pre Med* 2013;30:225–31.
17. Hyland A, Laux FL, Higbee C, et al. Cigarette purchase patterns in four countries and the relationship with cessation: findings from the International Tobacco Control (ITC) Four Country Survey. *Tob Res* 2006;15(Suppl III):iii59–iii64.
18. Licht AS, Hyland AJ, O'Connor RJ, et al. How do price minimizing behaviors impact smoking cessation? findings from the International Tobacco Control (ITC) Four Country Survey. *Int J Environ Res Public Health* 2011;8(5):1671–91.
19. Choi K, Hennrikus DJ, Forster JL, et al. Receipt and redemption of cigarette coupons, perceptions of cigarette companies and smoking cessation. *Tob Control* 2013; 22(6): 418-22.

For peer review only - http://bmjopen.bmj.com/site/about/guidelines.xhtml

20. Choi K, Forster JL, Erickson D, et al. The reciprocal relationships between changes in adolescent perceived prevalence of smoking in movies and progression of smoking status. *Tob Control.* 2012; 21(5):492-6
21. Maxwell JC. *The Maxwell Report: Year End & Fourth Quarter 2010 Sales Estimates for the Cigarette Industry*. Richmond (VA): John C. Maxwell, Jr., 2011 (accessed 24 Apr 2012).
22. Congressional Budget Office. *Raising the excise tax on cigarettes: Effects on health and the federal budget*. Washington: Congressional Budget Office, 2012. CBO Publication No. 4036.
23. DeCicca P, Kenkel DS, Liu F. Excise tax avoidance: the case of state cigarette taxes. *J Health Econ* 2013; 32: 1130-1141.
24. Centers for Disease Control and Prevention, Consumption of cigarettes and combustible tobacco- United States, 2000–2011, *Morb Mortal Wkly Rep,* 2012; 61,30, 565–569.
25. Sfekas A, Lillard DR. Do Firms Use Coupons and In-store Discounts to Strategically Market Experience Goods Over the Consumption Life-Cycle? The Case of Cigarettes. NBER Working Paper Series no 19310.

For peer review only

Table 1. The use of in-store coupons or other price-related discounts by major cigarette manufactures†

	PM	RJR	LORILLARD (Newport only)	Other Companies[a]
Prevalence of brand use	43.4%	23.9%	15.0%	17.7%
Prevalence of coupons or discounts used by smokers of that manufacture	24.4%	21.0%	13.7%	11.1%
Price reduction per pack for smokers of that manufacture($)	-0.29**	-0.29**	-0.24*	-0.23
Average price per pack for smokers of that manufacture ($)	5.06	4.63	4.75	3.94
Percentage of discount rendered to smokers of that manufacture	5.7%	6.3%	5.1%	5.8%
N	4,850	3,274	960	2,682

Notes: N represents un-weighted sample size. All estimates were obtained with post-stratification weights. In regressions, the dependent variable is price paid per pack, and the variable of interest is whether using coupons, rebates, buy-1-get-1-free, 2-for-1, or any other special promotions for cigarettes in the most recent purchase. Control variables include demographic characteristics (race, age, gender, education, marital status, and employment status), state dummy variables, whether the respondent is a daily smoker, time to first cigarette since wake up, and all other price-minimization behaviors (i.e., purchase of generic brands, purchase of cartons, purchase on Indian reservation, or purchase through the Internet). Percentage of discount rendered is obtained by dividing average price per pack with price reduction associated with coupons and other price-related discounts.

†The self-reported use of coupons or other price-related discounts only reflects direct-to-consumer discounts from the industry. Therefore, industry's promotional allowances directly paid to retailers include or wholesalers are not included.

[a] Users of Sonoma, USA Gold brands, and other brand names which not listed in the survey.

**Statistically significant at 5% level; * statistically significant at 10% level.

For peer review only

Table 2. The use of in-store coupons or other price-related discounts by cigarette manufacturer and by top 10 leading U.S. brands[†]

		Prevalence of coupon use	% of discount	Average price	Price after discount	Rank[a]
PM	Basic	22.2%	1.1%	$4.41	$4.36	3
	Marlboro	25.6%	5.6%	$5.00	$4.72**	2
	Virginia Slims	10.6%	3.4%	$6.49	$6.27	9
RJR	Camel	33.9%	7.8%	$5.24	$4.83**	1
	Doral	15.6%	12.7%	$3.93	$3.43**	6
	Kool	11.6%	17.3%	$4.23	$3.50**	8
	Pall Mall	15.9%	1.3%	$3.19	$3.15	5
	Salem	10.5%	15.7%	$5.11	$4.31**	10
	Winston	17.1%	3.7%	$4.07	$3.92	4
Lorillard	Newport	13.7%	5.1%	$4.75	$4.51*	7

Notes: N represents un-weighted sample size. All estimates were obtained with post-stratification weights. In regressions, the dependent variable is price paid per pack, and the variable of interest is whether using coupons, rebates, buy-1-get-1-free, 2-for-1, or any other special promotions for cigarettes in the most recent purchase. Control variables include demographic characteristics (race, age, gender, education, marital status, and employment status), state dummy variables, whether the respondent is a daily smoker, time to first cigarette since wake up, and all other price-minimization behaviors (i.e., purchase of generic brands, purchase of cartons, purchase on Indian reservation, or purchase through the Internet). Percentage of discount rendered is obtained by dividing average price per pack with price reduction associated with coupons and other price-related discounts.

[†]The self-reported use of coupons or other price-related discounts only reflects direct-to-consumer discounts from the industry. Therefore, industry's promotional allowances directly paid to retailers include or wholesalers are not included.

[a] Based on prevalence of coupon use.

**Price reduction associated with coupons and other price-related discounts is statistically significant at 5% level, * statistically significant at 10% level.

Figure 1. Brand preference among U.S. adult smokers (2009-2010 NATS)

For peer review only

Can you refuse these discounts? An analysis of price-related promotions by U.S. cigarette companies

Ralph S. Caraballo, PhD[1], Xu Wang, PhD[1], Xin Xu, PhD[1]

[1]National Center for Chronic Disease Prevention and Health Promotion, Office on Smoking and Health, Centers for Disease Control and Prevention, Atlanta, GA

Corresponding Author:

Ralph S. Caraballo, PhD
Office on Smoking and Health
National Center for Chronic Disease Prevention and Health Promotion
Centers for Disease Control and Prevention
4770 Buford Highway, NE, MS K-50
Atlanta, Georgia 30341-3717
Phone: 770-488-5723
Fax: 770-488-5767
E-mail: rfc8@cdc.gov

The findings and conclusions in this report are those of the authors and do not necessarily represent the official position of the Centers for Disease Control and Prevention

Targeted journal: **British Medical Journal (BMJ) Open Access**
Abstract: Word count: (journal-recommended maximum of 300 words) is ~~294~~296
Word count: (journal-recommended maximum of 4,000 words) is ~~2,819~~3,035
~~21~~ 25 references (25 allowed)
2 tables and 1 figure (5 total allowed)

For peer review only

ABSTRACT (word limit ~~294~~296/300):

Objectives: Raising unit price of cigarette has been shown to be one of the most effective ways of reducing cigarette consumption and increase rates of successful quitting. However, researches have shown that price-sensitive smokers have used a variety of strategies to mitigate the effect of rising price of cigarettes on their smoking habits. In particular, 23%-34% of adult smokers in the U.S. use cheaper brands, and 18%-55% use coupons or promotions. Although some studies have assessed smokers' social-demographic characteristics by their preferences to generic brands or premium brands, little is known about the discount use by type of brands. As such, the main purpose of this analysis is to evaluate the uses and price discount effects of these price-related discounts by manufacturer and major brand.

Formatted: Highlight

Setting: An analysis based on the cross-sectional 2009-2010 National Adult Tobacco Survey (NATS).

Participants: 11,766 current smokers aged 18 or above in the U.S.

Primary outcome measures: Price-related discount was defined as smokers who used coupons, rebates, buy 1 get 1 free, 2 for 1, or any other special promotions for their last cigarettes purchase.

Results: The use of price-related discounts and associated price impact vary widely by cigarette manufacturer and brand. Approximately 1 of 3 Camel, 1 of 4 Marlboro, and 1 of 8 Newport smokers used price-related discounts in their latest cigarette purchases. The average price reductions of discounts offered by Philip Morris (PM) or R.J. Reynolds (RJR) were around 29 cents per pack while that of Lorillard (Newport only) was 24 cents per pack. Cigarette brands that provided significant per pack price reductions include: PM Marlboro (28 cents), RJR brands Camel (41 cents), Doral (50 cents), Kool (73 cents), and Salem (80 cents), and Lorillard Newport (24 cents).

For peer review only

For peer review only - http://bmjopen.bmj.com/site/about/guidelines.xhtml

Conclusion: Policies that decrease price-minimization strategies will benefit public health.

For peer review only

For peer review only - http://bmjopen.bmj.com/site/about/guidelines.xhtml

Cigarette use is the most preventable cause of death and disease in the United States and presents a significant public health burden.[1] Research has shown that increasing the unit price of cigarettes is among the most effective public health interventions to reduce cigarette consumption, prevent smoking initiation, and increase rates of successful quitting.[2-7] In addition, recent evidence shows that the 2009 federal tobacco excise tax increases have been one of the strategies that have substantially reduced the number of cigarette and smokeless tobacco users among U.S. middle and high school students.[8]

Internal documents from cigarette companies have shown that cigarette companies are aware of the potential impacts that price increases have on their sales and profits. Cigarette companies have developed a variety of price-reduction marketing efforts to promote cigarette sales, such as multipack discounts, rebates, and coupons.[9] According to the most recent cigarette report from the Federal Trade Commission, in addition to giving away 50 million cigarettes for free in 2010, the major cigarette manufactures spent approximately $8.05 billion marketing their products. More than 80% of the marketing expenditures (6.49 billion) went to price-related discounts and promotional allowances used to reduce the retail price of cigarettes.[10]

These cigarette companies' price-related discounts may diminish the public health benefit associated with increased cigarette prices among some smokers even after federal law has raised the unit price of cigarettes. Several recent studies have shown that a large portion of U.S. adult smokers (18%–55%) have taken advantage of these price-related discounts offered by some cigarette companies.[11-16] In addition, evidence from other studies has shown that smokers who used these price-related discounts were less likely to make quit attempts or to successfully quit in the future.[17-20] Although studies have previously investigated demographics and socioeconomic characteristics of smokers who used price minimization strategies, including using coupons or

For peer review only - http://bmjopen.bmj.com/site/about/guidelines.xhtml

other types of discounts from cigarette companies,[11-15] little is known about how these price-related discounts affect the average price paid per cigarette when factoring in discounts offered by specific cigarette companies or when looking at specific cigarette brands. Cigarette companies may be directly influencing the prices of their products by using these types of marketing strategies.

Using unique data from the 2009–2010 National Adult Tobacco Survey (NATS) about cigarette brands and price-related discounts used by adult smokers, we evaluated the uses and price discount effects of these price-minimization strategies by cigarette manufacturer and major brand. To the best of our knowledge, this is the first study to provide these estimates from a national representative sample of U.S. adult smokers. The findings of the analysis may help policy and public health stakeholders to further understand the promotion strategies of leading U.S. cigarette companies.

METHODS

Data source

The 2009–2010 NATS is a stratified, national, landline and cell phone survey conducted during October 2009–June 2010. The survey population is a representative sample of non-institutionalized adults aged 18 years or older at both state and national levels. The survey was developed by the Office on Smoking and Health at the Centers for Disease Control and Prevention and was designed primarily to assess the prevalence of tobacco use and the factors related to tobacco use among U.S. adults. The survey has 130 questions that provide information about demographics, health status, cigarette smoking behaviors, price minimization behaviors, cigarette brands preference, the use of other tobacco products, and smoking quit attempts. The

For peer review only - http://bmjopen.bmj.com/site/about/guidelines.xhtml

2009–2010 NATS completed a total of 118,581 interviews, including 110,634 by landline and 7,947 by cell phone. Because samples used for this analysis contain only de-identified observations, this research did not involve human subjects, as defined by Title 45 Code of Federal Regulations, Part 46, and institutional review board (IRB) approval was not required.

This analysis is restricted to current smokers who reported the cigarette brand name that they smoked most often during the past 30 days (n=16,015). Current smokers were defined as those who reported smoking at least 100 cigarettes in their lifetime and currently smoked every day or some days (n=16,542). Among them, respondents who failed to report a brand name were excluded (n=523). Because of the concern of small sample size (n=4), respondents who smoked Forsyth, which is a private brand label, were also excluded from the analysis.

In addition, respondents who failed to provide information on price paid for their latest purchase (n=978), the use of price-minimization strategies (n=2,794), demographic characteristics (age, race/ethnicity, gender, education, marital status, or employment status) (n=477), or time to first cigarette since wake up, were also excluded. The final sample size is 11,766.

Measures of brands and companies

In the survey, respondents were asked about the cigarette brand that they used most often during the past 30 days. A total of 17 brand choices were listed. Except Forsyth and the choice of other brands (n=3,299), the remaining 15 brand names are categorized as premium brands or generic brands. Premium brand names include Camel, Kool, Marlboro, Newport, Pall Mall, Parliament, Salem, Virginia Slims and Winston, and generic brands include Basic, Doral, GPC, Misty, Sonoma, and USA Gold.

For peer review only - http://bmjopen.bmj.com/site/about/guidelines.xhtml

To evaluate price-related discounts and promotions used by major companies, three major cigarette companies were identified on the basis of the 15 brand names above. They are Philip Morris (PM), R.J. Reynolds (RJR), and Lorillard. These companies jointly represented approximately 85% of total U.S. cigarette sales in 2010.[21] PM's brand names include Basic, Marlboro, Parliament, and Virginia Slims. Camel, Doral, GPC, Kool, Misty, Pall Mall, Salem, and Winston are brand manufactured by RJR. Lorillard has the brand Newport. The remaining brands, including Sonoma, USA Gold, and the choice of others brands, are included for other cigarette companies.

Measures of prices and discounts from the industry

The 2009–2010 NATS contains two types of price data. Current smokers who bought cigarettes by packs in their latest purchases were asked to report price paid per pack (after discounts or coupons) in dollars. Those who bought cigarettes by cartons were asked to report price paid per carton. Consequently, price per carton was divided by 10 to obtain a consistent measure of price paid per pack.

In the survey, current smokers were also asked whether they had taken advantage of coupons, rebates, buy-1-get-1-free, 2-for-1, or any other special promotions for cigarettes during the most recent purchase. These coupon and other discount offers were defined in the analysis as coupons and price-related discounts from the industry. Positive responses to this question were used to estimate the prevalence of usage of price-related discounts and promotions by manufacturer and brand name.

For peer review only - http://bmjopen.bmj.com/site/about/guidelines.xhtml

Statistical analysis

Cigarette prices reported in the 2009–2010 NATS may reflect the price paid by a smoker after using multiple price minimization strategies. To assess the independent price reduction associated with coupons and other price-related discounts directly from the industry, for each cigarette company or brand, regression analysis with the following specification was used to obtain adjusted average prices per pack.

$$Per\ pack\ price\ paid = \beta_1 + \beta_2 discounts + \beta_3 OtherPMS + \beta_4 Ciguse + \beta_5 Demographics + \beta_6 state$$

The dependent variable was per pack price paid for cigarettes. The key independent variable was the dichotomous indicator of using a coupon and other price-related discounts during the most recent purchase (discounts). The covariates of other price minimization strategies (otherPMS) included the use of premium or generic brands in the past 30 days, purchase of latest cigarette by carton or by pack, purchase on Indian reservations during the previous year, and purchase through the Internet during the previous year. Daily smoking and time to first cigarette of the day (ciguse) were included in regression analysis as measures of smoking intensity and nicotine dependence so as to control for other price minimization strategies that are not included in the survey, because heavy or more addicted smokers are more likely to use price minimization strategies.[11,12,14,15] Gender, age, race/ethnicity, education, marital and employment status (demographics), and state dummy indicators (state) were also included to account for individual differences and policy variations across states.

Thus, the constant, β_1, presents the adjusted average per pack price before using any price minimization strategies, and the coefficient, β_2 reflects the price reduction associated with price-related discounts directly from the industry. All analyses were performed using STATA

Formatted: Font: Highlight

Formatted: Font: Highlight

Formatted: Font: Highlight

Formatted: Font: Highlight

Formatted: Font: Highlight

Formatted: Font: Highlight

Formatted: Font: Highlight

For peer review only - http://bmjopen.bmj.com/site/about/guidelines.xhtml

(version 13). and Poststratified sampling weights were incorporated in all analyses to account for the complex survey design of the 2009-2010 NATS and nonresponse.weighted using national NATS weights.

RESULTS

Overall, among 11,766 adult current smokers, 38.4% identified Marlboro as the brand they used most often (Figure 1), followed by Newport (15.1%) and Camel (8.7%). The percentage of users of other identified brand names were all less than 5%, respectively, ranging from Pall Mall (4.9%) to GPC (0.5%). The combined remaining 15.2% of smokers usually smoked cigarette brands (classified as other brands) that were not identified in the 2009–2010 NATS.

Table 1 presents the use of coupons or other price-related discounts among adult smokers by cigarette manufacturer. Specifically, 43.4% (4,850) reported usually smoking cigarettes produced by PM, 23.9% (3,274) usually smoked cigarettes from RJR, and 15.0% (960) usually smoked Newport cigarettes (Lorillard) (Table 1). The remaining 17.7% (2,682) smoked cigarettes from other companies, including Sonoma and USA Gold, which do not belong to the three major cigarette companies identified in the analysis. Approximately 24.4% of U.S. adult smokers who smoked PM brands used coupons or other price-related discount offers from the company during their most recent cigarette purchase, compared with 21.0% of those who smoked RJR brands, 13.7% of those who smoked Newport (Lorillard), and 11.1% of those who smoked brands from other cigarette companies.

For peer review only

After adjusting for the use of multiple price minimization strategies, respondents' demographic characteristics, smoking intensity, and state policy variations, the average per pack prices paid for cigarettes from PM, RJR, Newport (Lorillard), and other companies were $5.06, $4.63, $4.75, and $3.94, respectively. The average price reductions of coupons or other discounts offered by PM or RJR were statistically significant and similar in magnitude, 29 cents per pack. That of Newport (Lorillard) cigarettes was 24 cents per pack and was marginally significant.

Table 2 presents the use of coupons or other price-related discounts by specific brand (10 self-reported leading brands only) from the 3 leading cigarette manufacturing companies in the United States. Table 2 is ordered by PM brands first, followed by RJR brands, and last by Newport, the only Lorillard brand listed. The prevalence of using coupons or other price-related discounts varied from 33.9% (Camel, RJR), to 25.6% (Marlboro, PM), 13.7% (Newport, Lorillard), and 10.5% the lowest (Salem, RJR). Thus, about 1 of 3 Camel smokers used these strategies during the last time they bought their cigarettes, compared to about 1 of 4 Marlboro smokers, and about 1 of 8 Newport smokers. The percent price reductions due to use of coupons or discounts ranged from 1.1% (Basic) to 17.3% (Kool). Among these 10 leading U.S. cigarette brands, the average price paid per pack not using any price minimization strategies ranged from paying $3.19 (Pall Mall, RJR) to $6.49 (Virginia Slims, PM). After using coupons or other price-related discounts, the average prices paid for a pack of cigarettes of the top 3 selling brands were 28 cents less for Marlboro ($4.72 instead of $5.00), 41 cents less for Camel ($4.83 instead of $5.24) and 24 cents less for Newport ($4.51 instead of $4.75). Also, those who smoked Salem and used coupons or other price-related discounts saved 80 cents the last time they purchased it. Finally, among these 10 leading U.S. brands, users of Camel (RJR), Marlboro (PM), and Basic (PM) used price minimization strategies the most.

For peer review only

Discussion:

Coupons or other price-related discounts from PM, RJR, and Lorillard were used by 14%-25% of their consumers and have provided price reductions for the smokers who used specific brands. Coupons or other price-related discounts from other companies did not result in statistically significant price per pack reductions for their consumers. Therefore, after controlling for the use of other price minimization strategies and respondents' smoking intensity and nicotine addition, the 3 leading cigarette companies provided price reductions for their products through coupons or other price-related discounts.

To put the range of price reductions associated with coupons or other price-related discounts into context, the cigarette federal tax was $1.01 per pack starting April 1st, 2009, and the weighted average state cigarette excise tax rate was $ 1.17 per pack in 2010. These numbers imply that the coupon discounts from the three leading cigarette companies (about 24–29 cents) offset 23.8%–28.7% of the ~~public health~~price impacts from the federal tax or 11.0%–13.3% of the price impacts from the federal and state excise taxes combined to the smokers of cigarettes produced by these manufactures. As a result, these offers brought actual average prices down for users of specific brands, while~~.~~ the price reductions associated with these discounts were likely to increase the sales in these companies. For example, with a price elasticity of overall demand for cigarettes among adults at somewhere between -0.3 and -0.7,[22] ceteris paribus, these price discounts can be translated into 10.2-23.9 million packs of cigarette sales in 2010 for Marlboro, 3.0-6.6 million packs for Newport, and 1.8-4.1 million packs for Camel.[21]

, ~~Therefore, after controlling for the use of other price minimization strategies and respondents' smoking intensity and nicotine addition, the 3 leading cigarette companies provided price reductions for their products through coupons or other price-related discounts.~~

For peer review only - http://bmjopen.bmj.com/site/about/guidelines.xhtml

Although it is true that statistically significant or marginally statistically significant price reductions were observed for the 3 leading U.S. cigarette companies, significant reductions are brand specific. The significant reductions were observed only for Marlboro (U.S. leading brand), Camel (second U.S. leading brand), Kool, Doral and Salem, while marginally for Newport (third U.S. leading brand). Thus, PM, RJR, and Lorillard have concentrated their efforts to provide price discounts mainly to their bestselling brands. This might be one of the reasons that a previous analysis failed to identify significant price reductions associated with promotional offers.[12]

This study has some limitations. First, the study design is cross-sectional. Therefore, the study findings may be specific only to the period October 2009–June 2010. However, the study covers the entire United States, and there is a variety of prices as a function of brand smoked and other factors. Second, most variables in the analysis are collected from recent purchases (i.e., price paid, coupon and other price-related discounts, and carton purchase are for the latest purchase; premium or generic brands are for the last 30 days), but others are collected with a time frame of 1 year (i.e., purchase on Indian reservations, purchase through Internet). However, when we excluded Indian reservation and Internet purchases from the analysis, the adjusted average prices and price discounts associated with coupons did not change much. Third, because of an approval delay, only approximately 20% of respondents interviewed during the first 2 months of the survey were asked if purchases had been made on an Indian reservation anytime during the past year. In subsequent months, this question was asked of more than 90% of respondents. In the full sample, total missing responses for this question were 18.4% (2,770). However, sensitivity analysis has shown that dropping these observations does not significantly affect the results.[16] As noted in the method section, we also excluded respondents who failed to

For peer review only - http://bmjopen.bmj.com/site/about/guidelines.xhtml

report price paid for their latest purchase, the brand name they used most often in the last 30 days, or some of their demographic characteristics. We compared smoking and social-demographic characteristics between individuals with incomplete information and individuals with complete information and have found little differences at the mean level. 4,008 respondents were excluded from the analysis because they failed to provide price paid for their latest purchase, did not report the number of cigarettes smoked per day on smoking days, or failed to report using price minimization strategies. Only approximately 20% of respondents interviewed during the first 2 months of the survey were asked if purchases had been made on an Indian reservation anytime during the past year. However, another study has shown that dropping these observations does not significantly affect the results.[16] Fourth, as noted above, the 2009-2010 NATS does not collect information for all price minimization strategies, including cigarette purchases from states with lower price. Although the cross-border purchase is an issue in tobacco control, the prevalence of this behavior was quite low in the United States compared to other forms of price minimization strategies. For example, data from the 2003 and 2006-2007 Tobacco Use Supplement to the Current Population Survey (TUS-CPS) suggests that about 5% of smokers made purchase across a state border,[22] [23] while in the 2010-2011 TUS-CPS, approximately 3.0% of smokers purchased cigarettes from non-tribal land in lower-taxed and non-residential states (estimates not shown). In order to account for unmeasured price minimization strategies, we controlled for smoking intensity and level of nicotine addiction in the analysis since the literature suggests that these are important risk factors of using any price minimization strategies. Finally, the paid prices were determined by using self-reported information from the smoker, which may be subject to recall bias. However, existing evidence indicates that the average of self-reported prices per pack in the 2009-2010 NATS was very

Formatted: Font: Highlight

For peer review only - http://bmjopen.bmj.com/site/about/guidelines.xhtml

consistent with the corresponding 2009 national average price reported in the Tax Burden on Tobacco (TBOT).[16] Another benefit of using self-reported price in this analysis is that we are able to control for the corresponding smoking intensity of each smoker, which is not available in market scanned data but closely related to potential use of unmeasured price minimization strategies.

In addition to cigarette companies directly influencing cigarette retail prices by providing coupons or other price-related discounts, cigarette companies may also indirectly affect cigarette prices by offering discounts to retailers and by promoting cigars or pipe tobacco that can be used in roll-your-own cigarettes.~~[23]~~[24] Although these indirect influences are critical in tobacco control and certainly warrant additional studies, the NATS survey did not collect such information thus they are not within the scope of this analysis.

Our results show that the three leading cigarette companies in the United States continue to offer price discounts to smokers of their brands, although these promotions appear to be concentrated among their top-selling cigarette brands. Cigarette companies can be strategic when offering price discounts. For example, existing literature suggests that young adults, females, and heavy smokers are more frequently targeted for these promotions.[19] Other study have shown that cigarette brands with low market share target young adults with the goal of encouraging brand switching, while major brands target older smokers to facilitate brand loyalty.~~[24]~~[25] As pointed out earlier, smokers who use price-related discounts are less likely to make quit attempts or to successfully quit in the future. The length (duration) of smoking and the amount of cigarettes smoked per day on days the person smoked is strongly associated with a higher likelihood of developing and dying from a smoking-related disease, such as lung cancer, COPD, or heart attacks.[1] Therefore, even though price minimization strategies may increase sales and profits for

Formatted: Highlight

For peer review only

For peer review only - http://bmjopen.bmj.com/site/about/guidelines.xhtml

cigarette companies, these price discounts are likely preventing or delaying some smokers from permanent cessation. Policies that decrease price-minimization strategies will benefit public health.

For peer review only

For peer review only - http://bmjopen.bmj.com/site/about/guidelines.xhtml

References

1. United States Department of Health and Human Services. *The Health Consequences of Smoking: A Report of the Surgeon General*. Atlanta, GA: Centers for Disease Control and Prevention, 2004. http://www.cdc.gov/tobacco/data_statistics/sgr/2004/index.htm. (accessed 14 Dec 2012).
2. Chaloupka FJ, Straif K, and Leon ME. Effectiveness of tax and price policies in tobacco control. *Tob Control* 2011;20:235–8.
3. Chaloupka, FJ, Warner, KE. 2000. The economics of smoking. In Culyer,Anthony, and Joseph Newhouse (eds.), *The Handbook of Health Economics*, v.1B, 1539–1627. Elsevier, Amsterdam.
4. Institute of Medicine. *Ending the Tobacco Problem: A Blueprint for the Na*tion. Washington, DC: National Academies Press; 2007.
5. U.S. Department of Health and Human Services. *Reducing Tobacco Use: A Report of the Surgeon General*. Atlanta, Georgia: U.S. Department of Health and Human Services, Centers for Disease Control Prevention; 2000.
6. U.S. Department of Health and Human Services. *Preventing Tobacco Use Among Youth and Young Adults: a Report of the Surgeon General*. Atlanta, GA: US Department of Health and Human Services, Centers for Disease Control and Prevention; 2012.
7. Task Force on Community Preventive Services. Recommendations regarding interventions to reduce tobacco use and exposure to environmental tobacco smoke.*Am J Prev Med* 2001;20(2S):10–5.
8. Huang J, Chaloupka FJ. The impact of the 2009 Federal Tobacco Excise Tax increase on youth tobacco use. Cambridge, MA: National Bureau of Economic Research; 2012. NBER Working Paper no. 18026.
9. Chaloupka FJ, Cummings KM, Morley CP, Horan JK. Tax, price and cigarette smoking: evidence from the tobacco documents and implications for tobacco company marketing strategies. *Tob Control* 2002;11(Suppl I):i62-i72.
10. U.S. Federal Trade Commission. *Cigarette Report for 2009 and 2010*. Washington, DC: Federal Trade Commission; 2012. http://ftc.gov/os/2012/09/120921cigarettereport.pdf (accessed 4 Feb 2013).
11. Hyland A, Bauer JE, Li Q, Abrams SM, Higbee C, Peppone L, *et al*. Higher cigarette prices influence cigarette purchase patterns. *Tob Control* 2005;14:86–92.
12. White VM, Gilpin EA, White MM, Pierce JP. How do smokers control their cigarette expenditures? *Nicotine Tob Res* 2005;7:625–35.
13. White VM, White MM, Freeman K, Gilpin EA, Pierce JP. Cigarette promotional offers: who takes advantage? *Am J Pre Med* 2006;30:225–31.
14. Choi K, Hennrikus D, Forster J, St. Clair AW. Use of price-minimizing strategies by smokers and their effects on subsequent smoking behaviors. *Nicotine Tob Res* 2012;14(7):864–70.
15. Licht AS, Hyland AJ, O'Connor RJ, Chaloupka FJ, Borland R, Fong GT, *et al*. Socio-economic variation in price minimizing behaviors: findings from the International Tobacco Control (ITC) Four Country Survey. *Int J Environ Res Public Health* 2011;8:234–52.
16. Xu X, Pesko MF, Tynan MA, Gerzoff R, Malarcher AM, Pechacek TF. Cigarette price-minimizing strategies by U.S. smokers. *Am J Pre Med* 2013;30:225–31.
17. Hyland A, Laux FL, Higbee C, Hastings G, Ross H, *et al*. Cigarette purchase patterns in four countries and the relationship with cessation: findings from the International Tobacco Control (ITC) Four Country Survey. *Tob Res* 2006;15(Suppl III):iii59–iii64.
18. Licht AS, Hyland AJ, O'Connor RJ, Chaloupka FJ, Borland R, *et al*. How do price minimizing behaviors impact smoking cessation? findings from the International Tobacco Control (ITC) Four Country Survey. *Int J Environ Res Public Health* 2011;8(5):1671–91.
19. Choi K, Hennrikus DJ, Forster JL, Moilanen M. Receipt and redemption of cigarette coupons, perceptions of cigarette companies and smoking cessation. *Tob Control* 2013; 22(6): 418-22.

For peer review only

20. Choi K, Forster JL, Erickson D, Lazovich D, Southwell BG. The reciprocal relationships between changes in adolescent perceived prevalence of smoking in movies and progression of smoking status. *Tob Control.* 2012; 21(5):492-6
21. Maxwell JC. *The Maxwell Report: Year End & Fourth Quarter 2010 Sales Estimates for the Cigarette Industry*. Richmond (VA): John C. Maxwell, Jr., 2011 (accessed 24 Apr 2012).
22. Congressional Budget Office. *Raising the excise tax on cigarettes: Effects on health and the federal budget*. Washington: Congressional Budget Office, 2012. CBO Publication No. 4036.
23. DeCicca P, Kenkel DS, Liu F. Excise tax avoidance: the case of state cigarette taxes. *J Health Econ* 2013; 32: 1130-1141.
24. Centers for Disease Control and Prevention, Consumption of cigarettes and combustible tobacco-United States, 2000–2011, *Morb Mortal Wkly Rep,* 2012; 61,30, 565–569.
25. Sfekas A, Lillard DR. Do Firms Use Coupons and In-store Discounts to Strategically Market Experience Goods Over the Consumption Life-Cycle? The Case of Cigarettes. NBER Working Paper Series no 19310.

For peer review only

Figure 1. Brand preference among U.S. adult smokers (2009-2010 NATS)

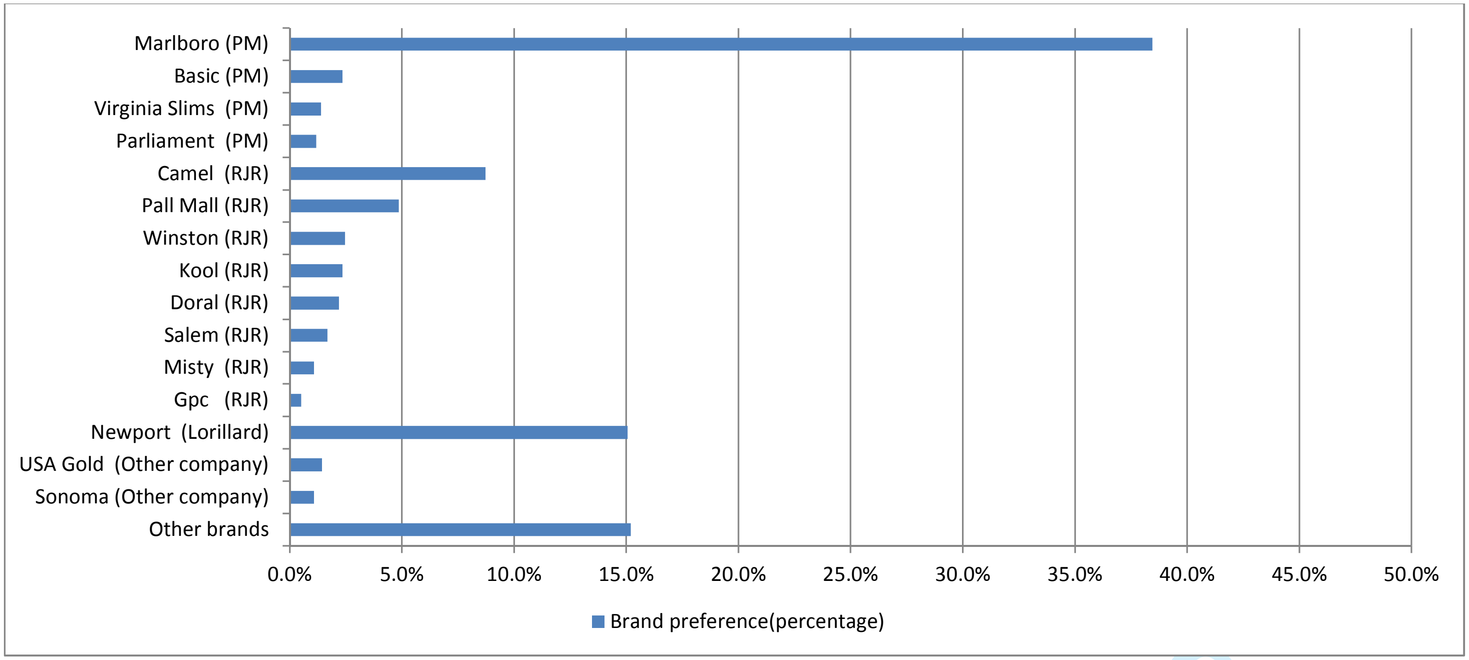

For peer review only - http://bmjopen.bmj.com/site/about/guidelines.xhtml

Table 1. The use of in-store coupons or other price-related discounts by major cigarette manufactures[†]

	PM	RJR	LORILLARD (Newport only)	Other Companies[a]
Prevalence of brand use	43.4%	23.9%	15.0%	17.7%
Prevalence of coupons or discounts used by smokers of that manufacture	24.4%	21.0%	13.7%	11.1%
Price reduction per pack for smokers of that manufacture($)	-0.29**	-0.29**	-0.24*	-0.23
Average price per pack for smokers of that manufacture ($)	5.06	4.63	4.75	3.94
Percentage of discount rendered to smokers of that manufacture	5.7%	6.3%	5.1%	5.8%
N	4,850	3,274	960	2,682

Notes: N represents un-weighted sample size. All estimates were obtained with poststratification~~national~~ weights. In regressions, the dependent variable is price paid per pack, and the variable of interest is whether using coupons, rebates, buy-1-get-1-free, 2-for-1, or any other special promotions for cigarettes in the most recent purchase. Control variables include demographic characteristics (race, age, gender, education, marital status, and employment status), state dummy variables, whether the respondent is a daily smoker, time to first cigarette since wake up, and all other price-minimization behaviors (i.e., purchase of generic brands, purchase of cartons, purchase on Indian reservation, or purchase through the Internet). Percentage of discount rendered is obtained by dividing average price per pack with price reduction associated with coupons and other price-related discounts.

[†]The self-reported use of coupons or other price-related discounts only reflects direct-to-consumer discounts from the industry. Therefore, industry's promotional allowances directly paid to retailers include or wholesalers are not included.

[a] Users of Sonoma, USA Gold brands, and other brand names which not listed in the survey.

[**]Statistically significant at 5% level; * statistically significant at 10% level.

For peer review only

Table 2. The use of in-store coupons or other price-related discounts by cigarette manufacturer and by top 10 leading U.S. brands[†]

		Prevalence of coupon use	% of discount	Average price	Price after discount	Rank[a]
PM	Basic	22.2%	1.1%	$4.41	$4.36	3
	Marlboro	25.6%	5.6%	$5.00	$4.72**	2
	Virginia Slims	10.6%	3.4%	$6.49	$6.27	9
RJR	Camel	33.9%	7.8%	$5.24	$4.83**	1
	Doral	15.6%	12.7%	$3.93	$3.43**	6
	Kool	11.6%	17.3%	$4.23	$3.50**	8
	Pall Mall	15.9%	1.3%	$3.19	$3.15	5
	Salem	10.5%	15.7%	$5.11	$4.31**	10
	Winston	17.1%	3.7%	$4.07	$3.92	4
Lorillard	Newport	13.7%	5.1%	$4.75	$4.51*	7

Notes: N represents un-weighted sample size. All estimates were obtained with poststratification weights. In regressions, the dependent variable is price paid per pack, and the variable of interest is whether using coupons, rebates, buy-1-get-1-free, 2-for-1, or any other special promotions for cigarettes in the most recent purchase. Control variables include demographic characteristics (race, age, gender, education, marital status, and employment status), state dummy variables, whether the respondent is a daily smoker, time to first cigarette since wake up, and all other price-minimization behaviors (i.e., purchase of generic brands, purchase of cartons, purchase on Indian reservation, or purchase through the Internet). Percentage of discount rendered is obtained by dividing average price per pack with price reduction associated with coupons and other price-related discounts.

[†] The self-reported use of coupons or other price-related discounts only reflects direct-to-consumer discounts from the industry. Therefore, industry's promotional allowances directly paid to retailers include or wholesalers are not included.

[a] Based on prevalence of coupon use.

**Price reduction associated with coupons and other price-related discounts is statistically significant at 5% level, * statistically significant at 10% level.

For peer review only - http://bmjopen.bmj.com/site/about/guidelines.xhtml

Figure 1. Brand preference among U.S. adult smokers (2009-2010 NATS)

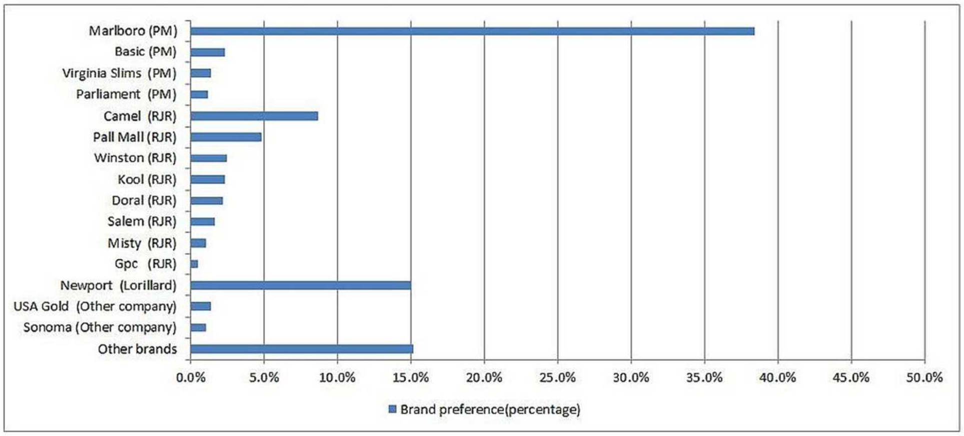

90x46mm (300 x 300 DPI)

BMJ Open

Can you refuse these discounts? An evaluation of the use and price discount impact of price-related promotions among U.S. adult smokers by cigarette manufacturer

Journal:	*BMJ Open*
Manuscript ID:	bmjopen-2013-004685.R2
Article Type:	Research
Date Submitted by the Author:	05-May-2014
Complete List of Authors:	Caraballo, Ralph; CDC, Office on Smoking and Health<br>Wang, Xu; CDC, Office on Smoking and Health<br>Xu, Xin; CDC, Office on Smoking and Health
**Primary Subject Heading**:	Smoking and tobacco
Secondary Subject Heading:	Health economics, Epidemiology
Keywords:	EPIDEMIOLOGY, HEALTH ECONOMICS, PUBLIC HEALTH

SCHOLARONE™
Manuscripts

For peer review only

Can you refuse these discounts? An evaluation of the use and price discount impact of price-related promotions among U.S. adult smokers by cigarette manufacturer

Ralph S. Caraballo, PhD[1], Xu Wang, PhD[1], Xin Xu, PhD[1]

[1]National Center for Chronic Disease Prevention and Health Promotion, Office on Smoking and Health, Centers for Disease Control and Prevention, Atlanta, GA

Corresponding Author:

Ralph S. Caraballo, PhD
Office on Smoking and Health
National Center for Chronic Disease Prevention and Health Promotion
Centers for Disease Control and Prevention
4770 Buford Highway, NE, MS K-50
Atlanta, Georgia 30341-3717
Phone: 770-488-5723
Fax: 770-488-5767
E-mail: rfc8@cdc.gov

The findings and conclusions in this report are those of the authors and do not necessarily represent the official position of the Centers for Disease Control and Prevention

Targeted journal: **British Medical Journal (BMJ) Open Access**
Abstract: Word count: (journal-recommended maximum of 300 words) is 296
Word count: (journal-recommended maximum of 4,000 words) is 3,035
25 references (25 allowed)
2 tables and 1 figure (5 total allowed)

For peer review only - http://bmjopen.bmj.com/site/about/guidelines.xhtml

ABSTRACT (word limit 300/300):

Objectives: Raising unit price of cigarette has been shown to be one of the most effective ways of reducing cigarette consumption and increase rates of successful quitting. However, researches have shown that price-sensitive smokers have used a variety of strategies to mitigate the effect of rising price of cigarettes on their smoking habits. In particular, 23%-34% of adult smokers in the U.S. use cheaper brands, and 18%-55% use coupons or promotions. Although some studies have assessed smokers' social-demographic characteristics by their preferences to generic brands or premium brands, little is known about the discount use by type of brands. As such, the main purpose of this analysis is to evaluate the uses and price discount effects of these price-related discounts by manufacturer and major brand.

Setting: An analysis based on the cross-sectional 2009-2010 National Adult Tobacco Survey (NATS).

Participants: 11,766 current smokers aged 18 or above in the U.S.

Primary outcome measures: Price-related discount was defined as smokers who used coupons, rebates, buy 1 get 1 free, 2 for 1, or any other special promotions for their last cigarettes purchase.

Results: The use of price-related discounts and associated price impact vary widely by cigarette manufacturer and brand. Approximately 1 of 3 Camel, 1 of 4 Marlboro, and 1 of 8 Newport smokers used price-related discounts in their latest cigarette purchases. The average price reductions of discounts offered by Philip Morris (PM) or R.J. Reynolds (RJR) were around 29 cents per pack while that of Lorillard (Newport only) was 24 cents per pack. Cigarette brands that provided significant per pack price reductions include: PM Marlboro (28 cents), RJR brands Camel (41 cents), Doral (50 cents), Kool (73 cents), and Salem (80 cents), and Lorillard Newport (24 cents).

For peer review only - http://bmjopen.bmj.com/site/about/guidelines.xhtml

For peer review only

Conclusion: Policies that decrease price-minimization strategies will benefit public health.

Strengths and limitations of this study:

Strengths:

- Research has shown that increasing the unit price of cigarettes is among the most effective public health interventions to reduce cigarette consumption, prevent smoking initiation, and increase rates of successful quitting. This is the first national study in the United States to evaluate the uses and effects of price-related discounts (coupons, rebates, buy 1 get 1 free, 2 for 1, or any other special promotions for the last pack of cigarettes purchased) by U.S. cigarette manufacturer and specific cigarette brand.

- The U.S. national study consisted of an analysis of telephone and cell phone cross sectional data (2009-2010) of 11,766 current cigarette smokers aged 18 or above.

- Price paid for last pack of cigarettes during the past 30 days was collected. Because of recent (last pack bought in past 30 days and most smokers are daily smokers), recall bias should not be a major problem in this study.

Limitations:

- The study design is cross-sectional. Therefore, the study findings may be specific only to the period October 2009–June 2010.

- .

- The 2009-2010 NATS does not collect information for all price minimization strategies, including cigarette purchases from states with lower price

- Smokers' self-reported use of coupon or other types of price-related discounts in the 2009-2010 NATS only reflects direct-to-consumer discounts from the industry. As a result, the discount measure in the analysis does not include tobacco industry's promotional allowances directly paid to cigarette retailers or wholesalers, as these disaggregated promotional allowances by cigarette manufacture or by brand have not been disclosed to consumers or to the public.

For peer review only - http://bmjopen.bmj.com/site/about/guidelines.xhtml

Cigarette use is the most preventable cause of death and disease in the United States and presents a significant public health burden.[1] Research has shown that increasing the unit price of cigarettes is among the most effective public health interventions to reduce cigarette consumption, prevent smoking initiation, and increase rates of successful quitting.[2-7] In addition, recent evidence shows that the 2009 federal tobacco excise tax increases have been one of the strategies that have substantially reduced the number of cigarette and smokeless tobacco users among U.S. middle and high school students.[8]

Internal documents from cigarette companies have shown that cigarette companies are aware of the potential impacts that price increases have on their sales and profits. Cigarette companies have developed a variety of price-reduction marketing efforts to promote cigarette sales, such as multipack discounts, rebates, and coupons.[9] According to the most recent cigarette report from the Federal Trade Commission, in addition to giving away 50 million cigarettes for free in 2010, the major cigarette manufactures spent approximately $8.05 billion marketing their products. More than 80% of the marketing expenditures (6.49 billion) went to price-related discounts and promotional allowances used to reduce the retail price of cigarettes.[10]

These cigarette companies' price-related discounts may diminish the public health benefit associated with increased cigarette prices among some smokers even after federal law has raised the unit price of cigarettes. Several recent studies have shown that a large portion of U.S. adult smokers (18%–55%) have taken advantage of these price-related discounts offered by some cigarette companies.[11-16] In addition, evidence from other studies has shown that smokers who used these price-related discounts were less likely to make quit attempts or to successfully quit in the future.[17-20] Although studies have previously investigated demographics and socioeconomic characteristics of smokers who used price minimization strategies, including using coupons or

For peer review only - http://bmjopen.bmj.com/site/about/guidelines.xhtml

other types of discounts from cigarette companies,[11-15] little is known about how these price-related discounts affect the average price paid per cigarette when factoring in discounts offered by specific cigarette companies or when looking at specific cigarette brands. Cigarette companies may be directly influencing the prices of their products by using these types of marketing strategies.

Using unique data from the 2009–2010 National Adult Tobacco Survey (NATS) about cigarette brands and price-related discounts used by adult smokers, we evaluated the uses and price discount effects of these price-minimization strategies by cigarette manufacturer and major brand. To the best of our knowledge, this is the first study to provide these estimates from a national representative sample of U.S. adult smokers. The findings of the analysis may help policy and public health stakeholders to further understand the promotion strategies of leading U.S. cigarette companies.

METHODS

Data source

The 2009–2010 NATS is a stratified, national, landline and cell phone survey conducted during October 2009–June 2010. The survey population is a representative sample of non-institutionalized adults aged 18 years or older at both state and national levels. The survey was developed by the Office on Smoking and Health at the Centers for Disease Control and Prevention and was designed primarily to assess the prevalence of tobacco use and the factors related to tobacco use among U.S. adults. The survey has 130 questions that provide information about demographics, health status, cigarette smoking behaviors, price minimization behaviors, cigarette brands preference, the use of other tobacco products, and smoking quit attempts. The

For peer review only

2009–2010 NATS completed a total of 118,581 interviews, including 110,634 by landline and 7,947 by cell phone. Because samples used for this analysis contain only de-identified observations, this research did not involve human subjects, as defined by Title 45 Code of Federal Regulations, Part 46, and institutional review board (IRB) approval was not required.

This analysis is restricted to current smokers who reported the cigarette brand name that they smoked most often during the past 30 days (n=16,015). Current smokers were defined as those who reported smoking at least 100 cigarettes in their lifetime and currently smoked every day or some days (n=16,542). Among them, respondents who failed to report a brand name were excluded (n=523). Because of the concern of small sample size (n=4), respondents who smoked Forsyth, which is a private brand label, were also excluded from the analysis.

In addition, respondents who failed to provide information on price paid for their latest purchase (n=978), the use of price-minimization strategies (n=2,794), demographic characteristics (age, race/ethnicity, gender, education, marital status, or employment status) (n=477), or time to first cigarette since wake up, were also excluded. The final sample size is 11,766.

Measures of brands and companies

In the survey, respondents were asked about the cigarette brand that they used most often during the past 30 days. A total of 17 brand choices were listed. Except Forsyth and the choice of other brands (n=3,299), the remaining 15 brand names are categorized as premium brands or generic brands. Premium brand names include Camel, Kool, Marlboro, Newport, Pall Mall, Parliament, Salem, Virginia Slims and Winston, and generic brands include Basic, Doral, GPC, Misty, Sonoma, and USA Gold.

For peer review only - http://bmjopen.bmj.com/site/about/guidelines.xhtml

To evaluate price-related discounts and promotions used by major companies, three major cigarette companies were identified on the basis of the 15 brand names above. They are Philip Morris (PM), R.J. Reynolds (RJR), and Lorillard. These companies jointly represented approximately 85% of total U.S. cigarette sales in 2010.[21] PM's brand names include Basic, Marlboro, Parliament, and Virginia Slims. Camel, Doral, GPC, Kool, Misty, Pall Mall, Salem, and Winston are brand manufactured by RJR. Lorillard has the brand Newport. The remaining brands, including Sonoma, USA Gold, and the choice of others brands, are included for other cigarette companies.

Measures of prices and discounts from the industry

The 2009–2010 NATS contains two types of price data. Current smokers who bought cigarettes by packs in their latest purchases were asked to report price paid per pack (after discounts or coupons) in dollars. Those who bought cigarettes by cartons were asked to report price paid per carton. Consequently, price per carton was divided by 10 to obtain a consistent measure of price paid per pack.

In the survey, current smokers were also asked whether they had taken advantage of coupons, rebates, buy-1-get-1-free, 2-for-1, or any other special promotions for cigarettes during the most recent purchase. These coupon and other discount offers were defined in the analysis as coupons and price-related discounts from the industry. Positive responses to this question were used to estimate the prevalence of usage of price-related discounts and promotions by manufacturer and brand name.

For peer review only - http://bmjopen.bmj.com/site/about/guidelines.xhtml

Statistical analysis

Cigarette prices reported in the 2009–2010 NATS may reflect the price paid by a smoker after using multiple price minimization strategies. To assess the independent price reduction associated with coupons and other price-related discounts directly from the industry, for each cigarette company or brand, regression analysis with the following specification was used to obtain adjusted average prices per pack.

$$Per\ pack\ price\ paid = \beta_1 + \beta_2 discounts + \beta_3 OtherPMS + \beta_4 Ciguse + \beta_5 Demographics + \beta_6 state$$

The dependent variable is per pack price paid for cigarettes. The key independent variable is the dichotomous indicator of using a coupon and other price-related discounts during the most recent purchase (discounts). The covariates of other price minimization strategies (OtherPMS) include four separate dichotomous variables, including the use of premium or generic brands in the past 30 days, purchase of latest cigarette by carton or by pack, purchase on Indian reservations during the previous year, and purchase through the Internet during the previous year. These variables are included to control for the possibility of using overlapping strategies during the latest cigarette purchase. Daily smoking and time to first cigarette of the day (ciguse) are included in regression analysis as measures of smoking intensity and nicotine dependence so as to control for other price minimization strategies that were not included in the survey, because heavy or more addicted smokers are more likely to use price minimization strategies.[11,12,14,15] Daily smoking is an indicator of whether or not the respondent was a daily smoker (versus some-daily smoker) at the time of the interview. Time to first cigarette after waking was a categorical variable of four (<5 minutes, 6-30 minutes, 31-60 minutes, and >60 minutes). Respondents' socio-demographic characteristics (demographics) and state dummy

For peer review only - http://bmjopen.bmj.com/site/about/guidelines.xhtml

indicators (state) are also included to account for individual difference and state policy variation. Assessed respondents' socio-demographics is a vector, which includes: gender (male or female); age group (18-25, 26-44, 45-64, and 65+ years); race/ethnicity (non-Hispanic White, non-Hispanic Black, Hispanic, non-Hispanic Asian, non-Hispanic Native Hawaiian/Pacific Islander, non-Hispanic American Indian/Alaska Native, and non-Hispanic "Other"); education (less than high school, high school graduate or equivalent, some college, and college degree or higher); marital status (married or cohabitate; widowed, divorced, or separated; and not currently in a relationship), and employment status (employed or unemployed).

Thus, the constant, β_1, presents the adjusted average per pack price before using any price minimization strategies, and the coefficient, β_2 reflects the price reduction associated with price-related discounts directly from the industry. All analyses were performed using STATA (version 13). Post-stratification sampling weights were incorporated in all analyses to account for the complex survey design of the 2009-2010 NATS and nonresponse.

RESULTS

Overall, among 11,766 adult current smokers, 38.4% identified Marlboro as the brand they used most often (Figure 1), followed by Newport (15.1%) and Camel (8.7%). The percentage of users of other identified brand names were all less than 5%, respectively, ranging from Pall Mall (4.9%) to GPC (0.5%). The combined remaining 15.2% of smokers usually smoked cigarette brands (classified as other brands) that were not identified in the 2009–2010 NATS.

For peer review only - http://bmjopen.bmj.com/site/about/guidelines.xhtml

Table 1 presents the use of coupons or other price-related discounts among adult smokers by cigarette manufacturer. Specifically, 43.4% (4,850) reported usually smoking cigarettes produced by PM, 23.9% (3,274) usually smoked cigarettes from RJR, and 15.0% (960) usually smoked Newport cigarettes (Lorillard) (Table 1). The remaining 17.7% (2,682) smoked cigarettes from other companies, including Sonoma and USA Gold, which do not belong to the three major cigarette companies identified in the analysis. Approximately 24.4% of U.S. adult smokers who smoked PM brands used coupons or other price-related discount offers from the company during their most recent cigarette purchase, compared with 21.0% of those who smoked RJR brands, 13.7% of those who smoked Newport (Lorillard), and 11.1% of those who smoked brands from other cigarette companies.

After adjusting for the use of multiple price minimization strategies, respondents' demographic characteristics, smoking intensity, and state policy variations, the average per pack prices paid for cigarettes from PM, RJR, Newport (Lorillard), and other companies were $5.06, $4.63, $4.75, and $3.94, respectively. The average price reductions of coupons or other discounts offered by PM or RJR were statistically significant and similar in magnitude, 29 cents per pack. That of Newport (Lorillard) cigarettes was 24 cents per pack and was marginally significant.

Table 2 presents the use of coupons or other price-related discounts by specific brand (10 self-reported leading brands only) from the 3 leading cigarette manufacturing companies in the United States. Table 2 is ordered by PM brands first, followed by RJR brands, and last by Newport, the only Lorillard brand listed. The prevalence of using coupons or other price-related discounts varied from 33.9% (Camel, RJR), to 25.6% (Marlboro, PM), 13.7% (Newport, Lorillard), and 10.5% the lowest (Salem, RJR). Thus, about 1 of 3 Camel smokers used these strategies during the last time they bought their cigarettes, compared to about 1 of 4 Marlboro

For peer review only - http://bmjopen.bmj.com/site/about/guidelines.xhtml

smokers, and about 1 of 8 Newport smokers. The percent price reductions due to use of coupons or discounts ranged from 1.1% (Basic) to 17.3% (Kool). Among these 10 leading U.S. cigarette brands, the average price paid per pack not using any price minimization strategies ranged from paying $3.19 (Pall Mall, RJR) to $6.49 (Virginia Slims, PM). After using coupons or other price-related discounts, the average prices paid for a pack of cigarettes of the top 3 selling brands were 28 cents less for Marlboro ($4.72 instead of $5.00), 41 cents less for Camel ($4.83 instead of $5.24) and 24 cents less for Newport ($4.51 instead of $4.75). Also, those who smoked Salem and used coupons or other price-related discounts saved 80 cents the last time they purchased it. Finally, among these 10 leading U.S. brands, users of Camel (RJR), Marlboro (PM), and Basic (PM) used price minimization strategies the most.

Discussion:

Coupons or other price-related discounts from PM, RJR, and Lorillard were used by 14%-25% of their consumers and have provided price reductions for the smokers who used specific brands. Coupons or other price-related discounts from other companies did not result in statistically significant price per pack reductions for their consumers. Therefore, after controlling for the use of other price minimization strategies and respondents' smoking intensity and nicotine addition, the 3 leading cigarette companies provided price reductions for their products through coupons or other price-related discounts.

To put the range of price reductions associated with coupons or other price-related discounts into context, the cigarette federal tax was $1.01 per pack starting April 1st, 2009, and the weighted average state cigarette excise tax rate was $ 1.17 per pack in 2010. These numbers

For peer review only - http://bmjopen.bmj.com/site/about/guidelines.xhtml

imply that the coupon discounts from the three leading cigarette companies (about 24–29 cents) offset 23.8%–28.7% of the price impacts from the federal tax or 11.0%–13.3% of the price impacts from the federal and state excise taxes combined to the smokers of cigarettes produced by these manufactures. As a result, these offers brought actual average prices down for users of specific brands, while the price reductions associated with these discounts were likely to increase the sales in these companies. For example, with a price elasticity of overall demand for cigarettes among adults at somewhere between -0.3 and -0.7,[22] ceteris paribus, these price discounts can be translated into 10.2-23.9 million packs of cigarette sales in 2010 for Marlboro, 3.0-6.6 million packs for Newport, and 1.8-4.1 million packs for Camel.[21]

Although it is true that statistically significant or marginally statistically significant price reductions were observed for the 3 leading U.S. cigarette companies, significant reductions are brand specific. The significant reductions were observed only for Marlboro (U.S. leading brand), Camel (second U.S. leading brand), Kool, Doral and Salem, while marginally for Newport (third U.S. leading brand). Thus, PM, RJR, and Lorillard have concentrated their efforts to provide price discounts mainly to their bestselling brands. This might be one of the reasons that a previous analysis failed to identify significant price reductions associated with promotional offers.[12]

This study has some limitations. First, the study design is cross-sectional. Therefore, the study findings may be specific only to the period October 2009–June 2010. However, the study covers the entire United States, and there is a variety of prices as a function of brand smoked and other factors. Second, most variables in the analysis are collected from recent purchases (i.e., price paid, coupon and other price-related discounts, and carton purchase are for the latest purchase; premium or generic brands are for the last 30 days), but others are collected with a

For peer review only - http://bmjopen.bmj.com/site/about/guidelines.xhtml

time frame of 1 year (i.e., purchase on Indian reservations, purchase through Internet). However, when we excluded Indian reservation and Internet purchases from the analysis, the adjusted average prices and price discounts associated with coupons did not change much. Third, because of an approval delay, only approximately 20% of respondents interviewed during the first 2 months of the survey were asked if purchases had been made on an Indian reservation anytime during the past year. In subsequent months, this question was asked of more than 90% of respondents. In the full sample, total missing responses for this question were 18.4% (3,503). However, sensitivity analysis has shown that dropping these observations does not significantly affect the results.[16] As noted in the method section, we also excluded respondents who failed to report price paid for their latest purchase, the brand name they used most often in the last 30 days, or some of their demographic characteristics. We compared smoking and social-demographic characteristics between individuals with incomplete information and individuals with complete information and have found little differences at the mean level. Fourth, as noted above, the 2009-2010 NATS does not collect information for all price minimization strategies, including cigarette purchases from states with lower price. Although the cross-border purchase is an issue in tobacco control, the prevalence of this behavior was quite low in the United States compared to other forms of price minimization strategies. For example, data from the 2003 and 2006-2007 Tobacco Use Supplement to the Current Population Survey (TUS-CPS) suggests that about 5% of smokers made purchase across a state border,[23] while in the 2010-2011 TUS-CPS, approximately 3.0% of smokers purchased cigarettes from non-tribal land in lower-taxed and non-residential states (estimates not shown). In order to account for unmeasured price minimization strategies, we controlled for smoking intensity and level of nicotine addiction in the analysis since the literature suggests that these are important risk factors of using any price

For peer review only - http://bmjopen.bmj.com/site/about/guidelines.xhtml

minimization strategies. Additionally, the paid prices were determined by using self-reported information from the smoker, which may be subject to recall bias. However, existing evidence indicates that the average of self-reported prices per pack in the 2009-2010 NATS was very consistent with the corresponding 2009 national average price reported in the Tax Burden on Tobacco (TBOT).[16] Another benefit of using self-reported price in this analysis is that we are able to control for the corresponding smoking intensity of each smoker, which is not available in market scanned data but closely related to potential use of unmeasured price minimization strategies. Finally, smokers' self-reported use of coupon or other types of price-related discounts in the 2009-2010 NATS only reflects direct-to-consumer discounts from the industry. As a result, the discount measure in the analysis does not include tobacco industry's promotional allowances directly paid to cigarette retailers or wholesalers, as these disaggregated promotional allowances by cigarette manufacture or by brand have not been disclosed to consumers or to the public.

In addition to cigarette companies directly influencing cigarette retail prices by providing coupons or other price-related discounts, cigarette companies may also indirectly affect cigarette prices by offering discounts to retailers and by promoting cigars or pipe tobacco that can be used in roll-your-own cigarettes.[24] Although these indirect influences are critical in tobacco control and certainly warrant additional studies, the NATS survey did not collect such information thus they are not within the scope of this analysis.

Our results show that the three leading cigarette companies in the United States continue to offer price discounts to smokers of their brands, although these promotions appear to be concentrated among their top-selling cigarette brands. Cigarette companies can be strategic when offering price discounts. For example, existing literature suggests that young adults, females, and

For peer review only - http://bmjopen.bmj.com/site/about/guidelines.xhtml

heavy smokers are more frequently targeted for these promotions.[19] Other study have shown that cigarette brands with low market share target young adults with the goal of encouraging brand switching, while major brands target older smokers to facilitate brand loyalty.[25] As pointed out earlier, smokers who use price-related discounts are less likely to make quit attempts or to successfully quit in the future. The length (duration) of smoking and the amount of cigarettes smoked per day on days the person smoked is strongly associated with a higher likelihood of developing and dying from a smoking-related disease, such as lung cancer, COPD, or heart attacks.[1] Therefore, even though price minimization strategies may increase sales and profits for cigarette companies, these price discounts are likely preventing or delaying some smokers from permanent cessation. Policies that decrease price-minimization strategies will benefit public health.

For peer review only

For peer review only - http://bmjopen.bmj.com/site/about/guidelines.xhtml

Contributorship Statement

All 3 authors meet the authorship criteria:

Ralph Caraballo participated in 1)the conception and design of data; 2) drafting the article or revising it critically for important intellectual content; and 3) final approval of the version to be published.

Xu Wang participated in 1) the conception and analysis and interpretation of data; 2) revising it critically for important intellectual content; and 3) final approval of the version to be published.

Xin Xu participated in 1)the conception and design of data; 2) drafting the article or revising it critically for important intellectual content; and 3) final approval of the version to be published.

Competing Interests

None

Data Sharing Statement

No additional data available

For peer review only

References

1. United States Department of Health and Human Services. *The Health Consequences of Smoking: A Report of the Surgeon General*. Atlanta, GA: Centers for Disease Control and Prevention, 2004. http://www.cdc.gov/tobacco/data_statistics/sgr/2004/index.htm. (accessed 14 Dec 2012).
2. Chaloupka FJ, Straif K, and Leon ME. Effectiveness of tax and price policies in tobacco control. *Tob Control* 2011;20:235–8.
3. Chaloupka, FJ, Warner, KE. 2000. The economics of smoking. In Culyer,Anthony, and Joseph Newhouse (eds.), *The Handbook of Health Economics*, v.1B, 1539–1627. Elsevier, Amsterdam.
4. Institute of Medicine. *Ending the Tobacco Problem: A Blueprint for the Na*tion. Washington, DC: National Academies Press; 2007.
5. U.S. Department of Health and Human Services. *Reducing Tobacco Use: A Report of the Surgeon General*. Atlanta, Georgia: U.S. Department of Health and Human Services, Centers for Disease Control Prevention; 2000.
6. U.S. Department of Health and Human Services. *Preventing Tobacco Use Among Youth and Young Adults: a Report of the Surgeon General*. Atlanta, GA: US Department of Health and Human Services, Centers for Disease Control and Prevention; 2012.
7. Task Force on Community Preventive Services. Recommendations regarding interventions to reduce tobacco use and exposure to environmental tobacco smoke.*Am J Prev Med* 2001;20(2S):10–5.
8. Huang J, Chaloupka FJ. The impact of the 2009 Federal Tobacco Excise Tax increase on youth tobacco use. Cambridge, MA: National Bureau of Economic Research; 2012. NBER Working Paper no. 18026.
9. Chaloupka FJ, Cummings KM, Morley CP, et al. Tax, price and cigarette smoking: evidence from the tobacco documents and implications for tobacco company marketing strategies. *Tob Control* 2002;11(Suppl I):i62-i72.
10. U.S. Federal Trade Commission. *Cigarette Report for 2009 and 2010*. Washington, DC: Federal Trade Commission; 2012. http://ftc.gov/os/2012/09/120921cigarettereport.pdf (accessed 4 Feb 2013).
11. Hyland A, Bauer JE, Li Q, *et al*. Higher cigarette prices influence cigarette purchase patterns. *Tob Control* 2005;14:86–92.
12. White VM, Gilpin EA, White MM, et al. How do smokers control their cigarette expenditures? *Nicotine Tob Res* 2005;7:625–35.
13. White VM, White MM, Freeman K, et al. Cigarette promotional offers: who takes advantage? *Am J Pre Med* 2006;30:225–31.
14. Choi K, Hennrikus D, Forster J, et al. Use of price-minimizing strategies by smokers and their effects on subsequent smoking behaviors. *Nicotine Tob Res* 2012;14(7):864–70.
15. Licht AS, Hyland AJ, O'Connor RJ, et al. Socio-economic variation in price minimizing behaviors: findings from the International Tobacco Control (ITC) Four Country Survey. *Int J Environ Res Public Health* 2011;8:234–52.
16. Xu X, Pesko MF, Tynan MA, et al. Cigarette price-minimizing strategies by U.S. smokers. *Am J Pre Med* 2013;30:225–31.
17. Hyland A, Laux FL, Higbee C, et al. Cigarette purchase patterns in four countries and the relationship with cessation: findings from the International Tobacco Control (ITC) Four Country Survey. *Tob Res* 2006;15(Suppl III):iii59–iii64.
18. Licht AS, Hyland AJ, O'Connor RJ, et al. How do price minimizing behaviors impact smoking cessation? findings from the International Tobacco Control (ITC) Four Country Survey. *Int J Environ Res Public Health* 2011;8(5):1671–91.
19. Choi K, Hennrikus DJ, Forster JL, et al. Receipt and redemption of cigarette coupons, perceptions of cigarette companies and smoking cessation. *Tob Control* 2013; 22(6): 418-22.

For peer review only - http://bmjopen.bmj.com/site/about/guidelines.xhtml

20. Choi K, Forster JL, Erickson D, et al. The reciprocal relationships between changes in adolescent perceived prevalence of smoking in movies and progression of smoking status. *Tob Control.* 2012; 21(5):492-6
21. Maxwell JC. *The Maxwell Report: Year End & Fourth Quarter 2010 Sales Estimates for the Cigarette Industry*. Richmond (VA): John C. Maxwell, Jr., 2011 (accessed 24 Apr 2012).
22. Congressional Budget Office. *Raising the excise tax on cigarettes: Effects on health and the federal budget*. Washington: Congressional Budget Office, 2012. CBO Publication No. 4036.
23. DeCicca P, Kenkel DS, Liu F. Excise tax avoidance: the case of state cigarette taxes. *J Health Econ* 2013; 32: 1130-1141.
24. Centers for Disease Control and Prevention, Consumption of cigarettes and combustible tobacco- United States, 2000–2011, *Morb Mortal Wkly Rep,* 2012; 61,30, 565–569.
25. Sfekas A, Lillard DR. Do Firms Use Coupons and In-store Discounts to Strategically Market Experience Goods Over the Consumption Life-Cycle? The Case of Cigarettes. NBER Working Paper Series no 19310.

For peer review only

Table 1. The use of in-store coupons or other price-related discounts by major cigarette manufactures†

	PM	RJR	LORILLARD (Newport only)	Other Companies[a]
Prevalence of brand use	43.4%	23.9%	15.0%	17.7%
Prevalence of coupons or discounts used by smokers of that manufacture	24.4%	21.0%	13.7%	11.1%
Price reduction per pack for smokers of that manufacture($)	-0.29**	-0.29**	-0.24*	-0.23
Average price per pack for smokers of that manufacture ($)	5.06	4.63	4.75	3.94
Percentage of discount rendered to smokers of that manufacture	5.7%	6.3%	5.1%	5.8%
N	4,850	3,274	960	2,682

Notes: N represents un-weighted sample size. All estimates were obtained with post-stratification weights. In regressions, the dependent variable is price paid per pack, and the variable of interest is whether using coupons, rebates, buy-1-get-1-free, 2-for-1, or any other special promotions for cigarettes in the most recent purchase. Control variables include demographic characteristics (race, age, gender, education, marital status, and employment status), state dummy variables, whether the respondent is a daily smoker, time to first cigarette since wake up, and all other price-minimization behaviors (i.e., purchase of generic brands, purchase of cartons, purchase on Indian reservation, or purchase through the Internet). Percentage of discount rendered is obtained by dividing average price per pack with price reduction associated with coupons and other price-related discounts.

†The self-reported use of coupons or other price-related discounts only reflects direct-to-consumer discounts from the industry. Therefore, industry's promotional allowances directly paid to retailers include or wholesalers are not included.

[a] Users of Sonoma, USA Gold brands, and other brand names which not listed in the survey.

**Statistically significant at 5% level; * statistically significant at 10% level.

For peer review only

Table 2. The use of in-store coupons or other price-related discounts by cigarette manufacturer and by top 10 leading U.S. brands[†]

		Prevalence of coupon use	% of discount	Average price	Price after discount	Rank[a]
PM	Basic	22.2%	1.1%	$4.41	$4.36	3
	Marlboro	25.6%	5.6%	$5.00	$4.72**	2
	Virginia Slims	10.6%	3.4%	$6.49	$6.27	9
RJR	Camel	33.9%	7.8%	$5.24	$4.83**	1
	Doral	15.6%	12.7%	$3.93	$3.43**	6
	Kool	11.6%	17.3%	$4.23	$3.50**	8
	Pall Mall	15.9%	1.3%	$3.19	$3.15	5
	Salem	10.5%	15.7%	$5.11	$4.31**	10
	Winston	17.1%	3.7%	$4.07	$3.92	4
Lorillard	Newport	13.7%	5.1%	$4.75	$4.51*	7

Notes: N represents un-weighted sample size. All estimates were obtained with post-stratification weights. In regressions, the dependent variable is price paid per pack, and the variable of interest is whether using coupons, rebates, buy-1-get-1-free, 2-for-1, or any other special promotions for cigarettes in the most recent purchase. Control variables include demographic characteristics (race, age, gender, education, marital status, and employment status), state dummy variables, whether the respondent is a daily smoker, time to first cigarette since wake up, and all other price-minimization behaviors (i.e., purchase of generic brands, purchase of cartons, purchase on Indian reservation, or purchase through the Internet). Percentage of discount rendered is obtained by dividing average price per pack with price reduction associated with coupons and other price-related discounts.

[†]The self-reported use of coupons or other price-related discounts only reflects direct-to-consumer discounts from the industry. Therefore, industry's promotional allowances directly paid to retailers include or wholesalers are not included.

[a] Based on prevalence of coupon use.

**Price reduction associated with coupons and other price-related discounts is statistically significant at 5% level, * statistically significant at 10% level.

For peer review only

Can you refuse these discounts? An ~~analysis~~ evaluation of the use~~s~~ and price discount ~~effects~~impact of ~~the~~ price-related promotions among U.S. adult smokers by ~~U.S.~~ cigarette ~~companies~~manufacturer

Ralph S. Caraballo, PhD[1], Xu Wang, PhD[1], Xin Xu, PhD[1]

[1]National Center for Chronic Disease Prevention and Health Promotion, Office on Smoking and Health, Centers for Disease Control and Prevention, Atlanta, GA

Corresponding Author:

Ralph S. Caraballo, PhD
Office on Smoking and Health
National Center for Chronic Disease Prevention and Health Promotion
Centers for Disease Control and Prevention
4770 Buford Highway, NE, MS K-50
Atlanta, Georgia 30341-3717
Phone: 770-488-5723
Fax: 770-488-5767
E-mail: rfc8@cdc.gov

The findings and conclusions in this report are those of the authors and do not necessarily represent the official position of the Centers for Disease Control and Prevention

Targeted journal: **British Medical Journal (BMJ) Open Access**
Abstract: Word count: (journal-recommended maximum of 300 words) is 296
Word count: (journal-recommended maximum of 4,000 words) is 3,035
25 references (25 allowed)
2 tables and 1 figure (5 total allowed)

For peer review only
For peer review only - http://bmjopen.bmj.com/site/about/guidelines.xhtml

ABSTRACT (word limit 300/300):

Objectives: Raising unit price of cigarette has been shown to be one of the most effective ways of reducing cigarette consumption and increase rates of successful quitting. However, researches have shown that price-sensitive smokers have used a variety of strategies to mitigate the effect of rising price of cigarettes on their smoking habits. In particular, 23%-34% of adult smokers in the U.S. use cheaper brands, and 18%-55% use coupons or promotions. Although some studies have assessed smokers' social-demographic characteristics by their preferences to generic brands or premium brands, little is known about the discount use by type of brands. As such, the main purpose of this analysis is to evaluate the uses and price discount effects of these price-related discounts by manufacturer and major brand.

Setting: An analysis based on the cross-sectional 2009-2010 National Adult Tobacco Survey (NATS).

Participants: 11,766 current smokers aged 18 or above in the U.S.

Primary outcome measures: Price-related discount was defined as smokers who used coupons, rebates, buy 1 get 1 free, 2 for 1, or any other special promotions for their last cigarettes purchase.

Results: The use of price-related discounts and associated price impact vary widely by cigarette manufacturer and brand. Approximately 1 of 3 Camel, 1 of 4 Marlboro, and 1 of 8 Newport smokers used price-related discounts in their latest cigarette purchases. The average price reductions of discounts offered by Philip Morris (PM) or R.J. Reynolds (RJR) were around 29 cents per pack while that of Lorillard (Newport only) was 24 cents per pack. Cigarette brands that provided significant per pack price reductions include: PM Marlboro (28 cents), RJR brands Camel (41 cents), Doral (50 cents), Kool (73 cents), and Salem (80 cents), and Lorillard Newport (24 cents).

For peer review only

Conclusion: Policies that decrease price-minimization strategies will benefit public health.

Strengths and limitations of this study:

Comment [XX(1]: Are we asked to provide these?

Strengths:

- Research has shown that increasing the unit price of cigarettes is among the most effective public health interventions to reduce cigarette consumption, prevent smoking initiation, and increase rates of successful quitting. This is the first national study in the United States to evaluate the uses and effects of price-related discounts (coupons, rebates, buy 1 get 1 free, 2 for 1, or any other special promotions for the last pack of cigarettes purchased) by U.S. cigarette manufacturer and specific cigarette brand.
- The U.S. national study consisted of an analysis of telephone and cell phone cross sectional data (2009-2010) of 11,766 current cigarette smokers aged 18 or above.
- Price paid for last pack of cigarettes during the past 30 days was collected. Because of recent (last pack bought in past 30 days and most smokers are daily smokers), recall bias should not be a major problem in this study.

Limitations:

- The study design is cross-sectional. Therefore, the study findings may be specific only to the period October 2009–June 2010.
- ~~4,776008 respondents were excluded from the analysis because they failed to provide price paid for their latest purchase, did not report the number of cigarettes smoked per day on smoking days, or the brand name they used most often in the last 30 days , failed to report using the use of price minimization strategies, or some of their demographic characteristics~~.
- The 2009-2010 NATS does not collect information for all price minimization strategies, including cigarette purchases from states with lower price
- Smokers' self-reported use of coupon or other types of price-related discounts in the 2009-2010 NATS only reflects direct-to-consumer discounts from the industry. As a result, the discount measure in the analysis does not include tobacco industry's promotional allowances directly paid to cigarette retailers or wholesalers, as these disaggregated promotional allowances by cigarette manufacture or by brand have not been disclosed to consumers or to the public.

Formatted: Normal, Left, Indent: First line: 0", Space After: 10 pt, Line spacing: Multiple 1.15 li

Formatted: Font color: Auto

For peer review only - http://bmjopen.bmj.com/site/about/guidelines.xhtml

Cigarette use is the most preventable cause of death and disease in the United States and presents a significant public health burden.[1] Research has shown that increasing the unit price of cigarettes is among the most effective public health interventions to reduce cigarette consumption, prevent smoking initiation, and increase rates of successful quitting.[2-7] In addition, recent evidence shows that the 2009 federal tobacco excise tax increases have been one of the strategies that have substantially reduced the number of cigarette and smokeless tobacco users among U.S. middle and high school students.[8]

Internal documents from cigarette companies have shown that cigarette companies are aware of the potential impacts that price increases have on their sales and profits. Cigarette companies have developed a variety of price-reduction marketing efforts to promote cigarette sales, such as multipack discounts, rebates, and coupons.[9] According to the most recent cigarette report from the Federal Trade Commission, in addition to giving away 50 million cigarettes for free in 2010, the major cigarette manufactures spent approximately $8.05 billion marketing their products. More than 80% of the marketing expenditures (6.49 billion) went to price-related discounts and promotional allowances used to reduce the retail price of cigarettes.[10]

These cigarette companies' price-related discounts may diminish the public health benefit associated with increased cigarette prices among some smokers even after federal law has raised the unit price of cigarettes. Several recent studies have shown that a large portion of U.S. adult smokers (18%–55%) have taken advantage of these price-related discounts offered by some cigarette companies.[11-16] In addition, evidence from other studies has shown that smokers who used these price-related discounts were less likely to make quit attempts or to successfully quit in the future.[17-20] Although studies have previously investigated demographics and socioeconomic characteristics of smokers who used price minimization strategies, including using coupons or

For peer review only - http://bmjopen.bmj.com/site/about/guidelines.xhtml

other types of discounts from cigarette companies,[11-15] little is known about how these price-related discounts affect the average price paid per cigarette when factoring in discounts offered by specific cigarette companies or when looking at specific cigarette brands. Cigarette companies may be directly influencing the prices of their products by using these types of marketing strategies.

Using unique data from the 2009–2010 National Adult Tobacco Survey (NATS) about cigarette brands and price-related discounts used by adult smokers, we evaluated the uses and price discount effects of these price-minimization strategies by cigarette manufacturer and major brand. To the best of our knowledge, this is the first study to provide these estimates from a national representative sample of U.S. adult smokers. The findings of the analysis may help policy and public health stakeholders to further understand the promotion strategies of leading U.S. cigarette companies.

METHODS

Data source

The 2009–2010 NATS is a stratified, national, landline and cell phone survey conducted during October 2009–June 2010. The survey population is a representative sample of non-institutionalized adults aged 18 years or older at both state and national levels. The survey was developed by the Office on Smoking and Health at the Centers for Disease Control and Prevention and was designed primarily to assess the prevalence of tobacco use and the factors related to tobacco use among U.S. adults. The survey has 130 questions that provide information about demographics, health status, cigarette smoking behaviors, price minimization behaviors, cigarette brands preference, the use of other tobacco products, and smoking quit attempts. The

For peer review only - http://bmjopen.bmj.com/site/about/guidelines.xhtml

2009–2010 NATS completed a total of 118,581 interviews, including 110,634 by landline and 7,947 by cell phone. Because samples used for this analysis contain only de-identified observations, this research did not involve human subjects, as defined by Title 45 Code of Federal Regulations, Part 46, and institutional review board (IRB) approval was not required.

This analysis is restricted to current smokers who reported the cigarette brand name that they smoked most often during the past 30 days (n=16,015). Current smokers were defined as those who reported smoking at least 100 cigarettes in their lifetime and currently smoked every day or some days (n=16,542). Among them, respondents who failed to report a brand name were excluded (n=523). Because of the concern of small sample size (n=4), respondents who smoked Forsyth, which is a private brand label, were also excluded from the analysis.

In addition, respondents who failed to provide information on price paid for their latest purchase (n=978), the use of price-minimization strategies (n=2,794), demographic characteristics (age, race/ethnicity, gender, education, marital status, or employment status) (n=477), or time to first cigarette since wake up, were also excluded. The final sample size is 11,766.

Measures of brands and companies

In the survey, respondents were asked about the cigarette brand that they used most often during the past 30 days. A total of 17 brand choices were listed. Except Forsyth and the choice of other brands (n=3,299), the remaining 15 brand names are categorized as premium brands or generic brands. Premium brand names include Camel, Kool, Marlboro, Newport, Pall Mall, Parliament, Salem, Virginia Slims and Winston, and generic brands include Basic, Doral, GPC, Misty, Sonoma, and USA Gold.

For peer review only

For peer review only - http://bmjopen.bmj.com/site/about/guidelines.xhtml

To evaluate price-related discounts and promotions used by major companies, three major cigarette companies were identified on the basis of the 15 brand names above. They are Philip Morris (PM), R.J. Reynolds (RJR), and Lorillard. These companies jointly represented approximately 85% of total U.S. cigarette sales in 2010.[21] PM's brand names include Basic, Marlboro, Parliament, and Virginia Slims. Camel, Doral, GPC, Kool, Misty, Pall Mall, Salem, and Winston are brand manufactured by RJR. Lorillard has the brand Newport. The remaining brands, including Sonoma, USA Gold, and the choice of others brands, are included for other cigarette companies.

Measures of prices and discounts from the industry

The 2009–2010 NATS contains two types of price data. Current smokers who bought cigarettes by packs in their latest purchases were asked to report price paid per pack (after discounts or coupons) in dollars. Those who bought cigarettes by cartons were asked to report price paid per carton. Consequently, price per carton was divided by 10 to obtain a consistent measure of price paid per pack.

In the survey, current smokers were also asked whether they had taken advantage of coupons, rebates, buy-1-get-1-free, 2-for-1, or any other special promotions for cigarettes during the most recent purchase. These coupon and other discount offers were defined in the analysis as coupons and price-related discounts from the industry. Positive responses to this question were used to estimate the prevalence of usage of price-related discounts and promotions by manufacturer and brand name.

For peer review only - http://bmjopen.bmj.com/site/about/guidelines.xhtml

Statistical analysis

Cigarette prices reported in the 2009–2010 NATS may reflect the price paid by a smoker after using multiple price minimization strategies. To assess the independent price reduction associated with coupons and other price-related discounts directly from the industry, for each cigarette company or brand, regression analysis with the following specification was used to obtain adjusted average prices per pack.

$$Per\ pack\ price\ paid = \beta_1 + \beta_2 discounts + \beta_3 OtherPMS + \beta_4 Ciguse + \beta_5 Demographics + \beta_6 state$$

The dependent variable ~~was~~ is per pack price paid for cigarettes. The key independent variable ~~was~~ is the dichotomous indicator of using a coupon and other price-related discounts during the most recent purchase (discounts). The covariates of other price minimization strategies (OtherPMS) include~~d~~ four separate dichotomous variables, including ~~to indicate~~ the use of premium or generic brands in the past 30 days, purchase of latest cigarette by carton or by pack, purchase on Indian reservations during the previous year, and purchase through the Internet during the previous year. These variables are included to control for the possibility of using overlapping strategies during the latest cigarette purchase. Daily smoking and time to first cigarette of the day (ciguse) ~~were~~ are included in regression analysis as measures of smoking intensity and nicotine dependence so as to control for other price minimization strategies that ~~are~~ were not included in the survey, because heavy or more addicted smokers are more likely to use price minimization strategies.[11,12,14,15] Daily smoking is~~was~~ ~~measured by~~ an ~~dichotomous variable that~~ indicat~~es~~or of whether or not the respondent ~~is~~was a ~~current~~ daily smoker (versus ~~current non~~some-daily smoker) at the time of the interview. Time to first cigarette after waking was ~~measured by four dummy~~a categorical variable of four~~s~~ (<5 minutes, 6-30 minutes, 31-60

For peer review only

minutes, and >60 minutes). ~~Gender, age, race/ethnicity, education, marital and employment status (demographics),~~ Respondents' socio-demographic characteristics (demographics) and state dummy indicators (state) are~~were~~ also included to account for individual difference~~s~~ and state policy variation~~s across states~~. Assessed respondents' socio-demographics is a vector, which include~~d~~s: gender (male or female); age group (18-25, 26-44, 45-64, and 65+ years); race/ethnicity (non-Hispanic White, non-Hispanic Black, Hispanic, non-Hispanic Asian, non-Hispanic Native Hawaiian/Pacific Islander, non-Hispanic American Indian/Alaska Native, and non-Hispanic "Other"); education (less than high school, high school graduate or equivalent, some college, and college degree or higher); marital status (married or cohabitate; widowed, divorced, or separated; and not currently in a relationship), and employment status (employed or unemployed).

Formatted: Indent: First line: 0.5", Space After: 9 pt

Thus, the constant, β_1, presents the adjusted average per pack price before using any price minimization strategies, and the coefficient, β_2 reflects the price reduction associated with price-related discounts directly from the industry. All analyses were performed using STATA (version 13). Post-stratification sampling weights were incorporated in all analyses to account for the complex survey design of the 2009-2010 NATS and nonresponse.

RESULTS

Overall, among 11,766 adult current smokers, 38.4% identified Marlboro as the brand they used most often (Figure 1), followed by Newport (15.1%) and Camel (8.7%). The percentage of users of other identified brand names were all less than 5%, respectively, ranging

For peer review only

from Pall Mall (4.9%) to GPC (0.5%). The combined remaining 15.2% of smokers usually smoked cigarette brands (classified as other brands) that were not identified in the 2009–2010 NATS.

Table 1 presents the use of coupons or other price-related discounts among adult smokers by cigarette manufacturer. Specifically, 43.4% (4,850) reported usually smoking cigarettes produced by PM, 23.9% (3,274) usually smoked cigarettes from RJR, and 15.0% (960) usually smoked Newport cigarettes (Lorillard) (Table 1). The remaining 17.7% (2,682) smoked cigarettes from other companies, including Sonoma and USA Gold, which do not belong to the three major cigarette companies identified in the analysis. Approximately 24.4% of U.S. adult smokers who smoked PM brands used coupons or other price-related discount offers from the company during their most recent cigarette purchase, compared with 21.0% of those who smoked RJR brands, 13.7% of those who smoked Newport (Lorillard), and 11.1% of those who smoked brands from other cigarette companies.

After adjusting for the use of multiple price minimization strategies, respondents' demographic characteristics, smoking intensity, and state policy variations, the average per pack prices paid for cigarettes from PM, RJR, Newport (Lorillard), and other companies were $5.06, $4.63, $4.75, and $3.94, respectively. The average price reductions of coupons or other discounts offered by PM or RJR were statistically significant and similar in magnitude, 29 cents per pack. That of Newport (Lorillard) cigarettes was 24 cents per pack and was marginally significant.

Table 2 presents the use of coupons or other price-related discounts by specific brand (10 self-reported leading brands only) from the 3 leading cigarette manufacturing companies in the United States. Table 2 is ordered by PM brands first, followed by RJR brands, and last by Newport, the only Lorillard brand listed. The prevalence of using coupons or other price-related

For peer review only - http://bmjopen.bmj.com/site/about/guidelines.xhtml

discounts varied from 33.9% (Camel, RJR), to 25.6% (Marlboro, PM), 13.7% (Newport, Lorillard), and 10.5% the lowest (Salem, RJR). Thus, about 1 of 3 Camel smokers used these strategies during the last time they bought their cigarettes, compared to about 1 of 4 Marlboro smokers, and about 1 of 8 Newport smokers. The percent price reductions due to use of coupons or discounts ranged from 1.1% (Basic) to 17.3% (Kool). Among these 10 leading U.S. cigarette brands, the average price paid per pack not using any price minimization strategies ranged from paying $3.19 (Pall Mall, RJR) to $6.49 (Virginia Slims, PM). After using coupons or other price-related discounts, the average prices paid for a pack of cigarettes of the top 3 selling brands were 28 cents less for Marlboro ($4.72 instead of $5.00), 41 cents less for Camel ($4.83 instead of $5.24) and 24 cents less for Newport ($4.51 instead of $4.75). Also, those who smoked Salem and used coupons or other price-related discounts saved 80 cents the last time they purchased it. Finally, among these 10 leading U.S. brands, users of Camel (RJR), Marlboro (PM), and Basic (PM) used price minimization strategies the most.

Discussion:

Coupons or other price-related discounts from PM, RJR, and Lorillard were used by 14%-25% of their consumers and have provided price reductions for the smokers who used specific brands. Coupons or other price-related discounts from other companies did not result in statistically significant price per pack reductions for their consumers. Therefore, after controlling for the use of other price minimization strategies and respondents' smoking intensity and nicotine addition, the 3 leading cigarette companies provided price reductions for their products through coupons or other price-related discounts.

For peer review only

To put the range of price reductions associated with coupons or other price-related discounts into context, the cigarette federal tax was $1.01 per pack starting April 1st, 2009, and the weighted average state cigarette excise tax rate was $ 1.17 per pack in 2010. These numbers imply that the coupon discounts from the three leading cigarette companies (about 24–29 cents) offset 23.8%–28.7% of the price impacts from the federal tax or 11.0%–13.3% of the price impacts from the federal and state excise taxes combined to the smokers of cigarettes produced by these manufactures. As a result, these offers brought actual average prices down for users of specific brands, while the price reductions associated with these discounts were likely to increase the sales in these companies. For example, with a price elasticity of overall demand for cigarettes among adults at somewhere between -0.3 and -0.7,[22] ceteris paribus, these price discounts can be translated into 10.2-23.9 million packs of cigarette sales in 2010 for Marlboro, 3.0-6.6 million packs for Newport, and 1.8-4.1 million packs for Camel.[21]

Although it is true that statistically significant or marginally statistically significant price reductions were observed for the 3 leading U.S. cigarette companies, significant reductions are brand specific. The significant reductions were observed only for Marlboro (U.S. leading brand), Camel (second U.S. leading brand), Kool, Doral and Salem, while marginally for Newport (third U.S. leading brand). Thus, PM, RJR, and Lorillard have concentrated their efforts to provide price discounts mainly to their bestselling brands. This might be one of the reasons that a previous analysis failed to identify significant price reductions associated with promotional offers.[12]

This study has some limitations. First, the study design is cross-sectional. Therefore, the study findings may be specific only to the period October 2009–June 2010. However, the study covers the entire United States, and there is a variety of prices as a function of brand smoked and

For peer review only

other factors. Second, most variables in the analysis are collected from recent purchases (i.e., price paid, coupon and other price-related discounts, and carton purchase are for the latest purchase; premium or generic brands are for the last 30 days), but others are collected with a time frame of 1 year (i.e., purchase on Indian reservations, purchase through Internet). However, when we excluded Indian reservation and Internet purchases from the analysis, the adjusted average prices and price discounts associated with coupons did not change much. Third, because of an approval delay, only approximately 20% of respondents interviewed during the first 2 months of the survey were asked if purchases had been made on an Indian reservation anytime during the past year. In subsequent months, this question was asked of more than 90% of respondents. In the full sample, total missing responses for this question were 18.4% (3,503). However, sensitivity analysis has shown that dropping these observations does not significantly affect the results.[16] As noted in the method section, we also excluded respondents who failed to report price paid for their latest purchase, the brand name they used most often in the last 30 days, or some of their demographic characteristics. We compared smoking and social-demographic characteristics between individuals with incomplete information and individuals with complete information and have found little differences at the mean level. Fourth, as noted above, the 2009-2010 NATS does not collect information for all price minimization strategies, including cigarette purchases from states with lower price. Although the cross-border purchase is an issue in tobacco control, the prevalence of this behavior was quite low in the United States compared to other forms of price minimization strategies. For example, data from the 2003 and 2006-2007 Tobacco Use Supplement to the Current Population Survey (TUS-CPS) suggests that about 5% of smokers made purchase across a state border,[23] while in the 2010-2011 TUS-CPS, approximately 3.0% of smokers purchased cigarettes from non-tribal land in lower-taxed and

For peer review only

non-residential states (estimates not shown). In order to account for unmeasured price minimization strategies, we controlled for smoking intensity and level of nicotine addiction in the analysis since the literature suggests that these are important risk factors of using any price minimization strategies. FifthinallyAdditionally, the paid prices were determined by using self-reported information from the smoker, which may be subject to recall bias. However, existing evidence indicates that the average of self-reported prices per pack in the 2009-2010 NATS was very consistent with the corresponding 2009 national average price reported in the Tax Burden on Tobacco (TBOT).[16] Another benefit of using self-reported price in this analysis is that we are able to control for the corresponding smoking intensity of each smoker, which is not available in market scanned data but closely related to potential use of unmeasured price minimization strategies. Finally, smokers' self-reported use of coupon or other types of price-related discounts in the 2009-2010 NATS only reflects direct-to-consumer discounts from the industry. As a result, the discount measure in the analysis does not include tobacco industry's promotional allowances directly paid to cigarette retailers or wholesalers, as these disaggregated promotional allowances by cigarette manufacture or by brand have not been disclosed to consumers or to the public.

In addition to cigarette companies directly influencing cigarette retail prices by providing coupons or other price-related discounts, cigarette companies may also indirectly affect cigarette prices by offering discounts to retailers and by promoting cigars or pipe tobacco that can be used in roll-your-own cigarettes.[24] Although these indirect influences are critical in tobacco control and certainly warrant additional studies, the NATS survey did not collect such information thus they are not within the scope of this analysis.

For peer review only - http://bmjopen.bmj.com/site/about/guidelines.xhtml

Our results show that the three leading cigarette companies in the United States continue to offer price discounts to smokers of their brands, although these promotions appear to be concentrated among their top-selling cigarette brands. Cigarette companies can be strategic when offering price discounts. For example, existing literature suggests that young adults, females, and heavy smokers are more frequently targeted for these promotions.[19] Other study have shown that cigarette brands with low market share target young adults with the goal of encouraging brand switching, while major brands target older smokers to facilitate brand loyalty.[25] As pointed out earlier, smokers who use price-related discounts are less likely to make quit attempts or to successfully quit in the future. The length (duration) of smoking and the amount of cigarettes smoked per day on days the person smoked is strongly associated with a higher likelihood of developing and dying from a smoking-related disease, such as lung cancer, COPD, or heart attacks.[1] Therefore, even though price minimization strategies may increase sales and profits for cigarette companies, these price discounts are likely preventing or delaying some smokers from permanent cessation. Policies that decrease price-minimization strategies will benefit public health.

For peer review only - http://bmjopen.bmj.com/site/about/guidelines.xhtml

Contributorship Statement

All 3 authors meet the authorship criteria:

Ralph Caraballo participated in 1)the conception and design of data; 2) drafting the article or revising it critically for important intellectual content; and 3) final approval of the version to be published.

Xu Wang participated in 1) the conception and analysis and interpretation of data; 2) revising it critically for important intellectual content; and 3) final approval of the version to be published.

Xin Xu participated in 1)the conception and design of data; 2) drafting the article or revising it critically for important intellectual content; and 3) final approval of the version to be published.

Competing Interests

None

Data Sharing Statement

The raw and weighted data set are available to the public. A request can be made to the authors

For peer review only

For peer review only - http://bmjopen.bmj.com/site/about/guidelines.xhtml

References

1. United States Department of Health and Human Services. *The Health Consequences of Smoking: A Report of the Surgeon General*. Atlanta, GA: Centers for Disease Control and Prevention, 2004. http://www.cdc.gov/tobacco/data_statistics/sgr/2004/index.htm. (accessed 14 Dec 2012).
2. Chaloupka FJ, Straif K, and Leon ME. Effectiveness of tax and price policies in tobacco control. *Tob Control* 2011;20:235–8.
3. Chaloupka, FJ, Warner, KE. 2000. The economics of smoking. In Culyer,Anthony, and Joseph Newhouse (eds.), *The Handbook of Health Economics*, v.1B, 1539–1627. Elsevier, Amsterdam.
4. Institute of Medicine. *Ending the Tobacco Problem: A Blueprint for the Na*tion. Washington, DC: National Academies Press; 2007.
5. U.S. Department of Health and Human Services. *Reducing Tobacco Use: A Report of the Surgeon General*. Atlanta, Georgia: U.S. Department of Health and Human Services, Centers for Disease Control Prevention; 2000.
6. U.S. Department of Health and Human Services. *Preventing Tobacco Use Among Youth and Young Adults: a Report of the Surgeon General*. Atlanta, GA: US Department of Health and Human Services, Centers for Disease Control and Prevention; 2012.
7. Task Force on Community Preventive Services. Recommendations regarding interventions to reduce tobacco use and exposure to environmental tobacco smoke.*Am J Prev Med* 2001;20(2S):10–5.
8. Huang J, Chaloupka FJ. The impact of the 2009 Federal Tobacco Excise Tax increase on youth tobacco use. Cambridge, MA: National Bureau of Economic Research; 2012. NBER Working Paper no. 18026.
9. Chaloupka FJ, Cummings KM, Morley CP, et al. Tax, price and cigarette smoking: evidence from the tobacco documents and implications for tobacco company marketing strategies. *Tob Control* 2002;11(Suppl I):i62-i72.
10. U.S. Federal Trade Commission. *Cigarette Report for 2009 and 2010*. Washington, DC: Federal Trade Commission; 2012. http://ftc.gov/os/2012/09/120921cigarettereport.pdf (accessed 4 Feb 2013).
11. Hyland A, Bauer JE, Li Q, Abrams SM, Higbee C, Peppone L, *et al*. Higher cigarette prices influence cigarette purchase patterns. *Tob Control* 2005;14:86–92.
12. White VM, Gilpin EA, White MM, et al. How do smokers control their cigarette expenditures? *Nicotine Tob Res* 2005;7:625–35.
13. White VM, White MM, Freeman K, et al. Cigarette promotional offers: who takes advantage? *Am J Pre Med* 2006;30:225–31.
14. Choi K, Hennrikus D, Forster J, et al. Use of price-minimizing strategies by smokers and their effects on subsequent smoking behaviors. *Nicotine Tob Res* 2012;14(7):864–70.
15. Licht AS, Hyland AJ, O'Connor RJ, et al. Socio-economic variation in price minimizing behaviors: findings from the International Tobacco Control (ITC) Four Country Survey. *Int J Environ Res Public Health* 2011;8:234–52.
16. Xu X, Pesko MF, Tynan MA, et al. Cigarette price-minimizing strategies by U.S. smokers. *Am J Pre Med* 2013;30:225–31.
17. Hyland A, Laux FL, Higbee C, et al. Cigarette purchase patterns in four countries and the relationship with cessation: findings from the International Tobacco Control (ITC) Four Country Survey. *Tob Res* 2006;15(Suppl III):iii59–iii64.
18. Licht AS, Hyland AJ, O'Connor RJ, et al. How do price minimizing behaviors impact smoking cessation? findings from the International Tobacco Control (ITC) Four Country Survey. *Int J Environ Res Public Health* 2011;8(5):1671–91.
19. Choi K, Hennrikus DJ, Forster JL, et al. Receipt and redemption of cigarette coupons, perceptions of cigarette companies and smoking cessation. *Tob Control* 2013; 22(6): 418-22.

For peer review only

20. Choi K, Forster JL, Erickson D, et al. The reciprocal relationships between changes in adolescent perceived prevalence of smoking in movies and progression of smoking status. *Tob Control.* 2012; 21(5):492-6
21. Maxwell JC. *The Maxwell Report: Year End & Fourth Quarter 2010 Sales Estimates for the Cigarette Industry*. Richmond (VA): John C. Maxwell, Jr., 2011 (accessed 24 Apr 2012).
22. Congressional Budget Office. *Raising the excise tax on cigarettes: Effects on health and the federal budget*. Washington: Congressional Budget Office, 2012. CBO Publication No. 4036.
23. DeCicca P, Kenkel DS, Liu F. Excise tax avoidance: the case of state cigarette taxes. *J Health Econ* 2013; 32: 1130-1141.
24. Centers for Disease Control and Prevention, Consumption of cigarettes and combustible tobacco-United States, 2000–2011, *Morb Mortal Wkly Rep,* 2012; 61,30, 565–569.
25. Sfekas A, Lillard DR. Do Firms Use Coupons and In-store Discounts to Strategically Market Experience Goods Over the Consumption Life-Cycle? The Case of Cigarettes. NBER Working Paper Series no 19310.

For peer review only

Table 1. The use of in-store coupons or other price-related discounts by major cigarette manufactures[†]

	PM	RJR	LORILLARD (Newport only)	Other Companies[a]
Prevalence of brand use	43.4%	23.9%	15.0%	17.7%
Prevalence of coupons or discounts used by smokers of that manufacture	24.4%	21.0%	13.7%	11.1%
Price reduction per pack for smokers of that manufacture($)	-0.29**	-0.29**	-0.24*	-0.23
Average price per pack for smokers of that manufacture ($)	5.06	4.63	4.75	3.94
Percentage of discount rendered to smokers of that manufacture	5.7%	6.3%	5.1%	5.8%
N	4,850	3,274	960	2,682

Notes: N represents un-weighted sample size. All estimates were obtained with post-stratification weights. In regressions, the dependent variable is price paid per pack, and the variable of interest is whether using coupons, rebates, buy-1-get-1-free, 2-for-1, or any other special promotions for cigarettes in the most recent purchase. Control variables include demographic characteristics (race, age, gender, education, marital status, and employment status), state dummy variables, whether the respondent is a daily smoker, time to first cigarette since wake up, and all other price-minimization behaviors (i.e., purchase of generic brands, purchase of cartons, purchase on Indian reservation, or purchase through the Internet). Percentage of discount rendered is obtained by dividing average price per pack with price reduction associated with coupons and other price-related discounts.

[†]The self-reported use of coupons or other price-related discounts only reflects direct-to-consumer discounts from the industry. Therefore, industry's promotional allowances directly paid to retailers include or wholesalers are not included.

[a] Users of Sonoma, USA Gold brands, and other brand names which not listed in the survey.

**Statistically significant at 5% level; * statistically significant at 10% level.

For peer review only - http://bmjopen.bmj.com/site/about/guidelines.xhtml

Table 2. The use of in-store coupons or other price-related discounts by cigarette manufacturer and by top 10 leading U.S. brands[†]

		Prevalence of coupon use	% of discount	Average price	Price after discount	Rank[a]
PM	Basic	22.2%	1.1%	$4.41	$4.36	3
	Marlboro	25.6%	5.6%	$5.00	$4.72**	2
	Virginia Slims	10.6%	3.4%	$6.49	$6.27	9
RJR	Camel	33.9%	7.8%	$5.24	$4.83**	1
	Doral	15.6%	12.7%	$3.93	$3.43**	6
	Kool	11.6%	17.3%	$4.23	$3.50**	8
	Pall Mall	15.9%	1.3%	$3.19	$3.15	5
	Salem	10.5%	15.7%	$5.11	$4.31**	10
	Winston	17.1%	3.7%	$4.07	$3.92	4
Lorillard	Newport	13.7%	5.1%	$4.75	$4.51*	7

Notes: N represents un-weighted sample size. All estimates were obtained with post-stratification weights. In regressions, the dependent variable is price paid per pack, and the variable of interest is whether using coupons, rebates, buy-1-get-1-free, 2-for-1, or any other special promotions for cigarettes in the most recent purchase. Control variables include demographic characteristics (race, age, gender, education, marital status, and employment status), state dummy variables, whether the respondent is a daily smoker, time to first cigarette since wake up, and all other price-minimization behaviors (i.e., purchase of generic brands, purchase of cartons, purchase on Indian reservation, or purchase through the Internet). Percentage of discount rendered is obtained by dividing average price per pack with price reduction associated with coupons and other price-related discounts.

[†]The self-reported use of coupons or other price-related discounts only reflects direct-to-consumer discounts from the industry. Therefore, industry's promotional allowances directly paid to retailers include or wholesalers are not included.

[a] Based on prevalence of coupon use.

**Price reduction associated with coupons and other price-related discounts is statistically significant at 5% level, * statistically significant at 10% level.

Figure 1. Brand preference among U.S. adult smokers (2009-2010 NATS)

Comment [XX(2]: Xu, is our figure 1 also missing from the original file?

For peer review only - http://bmjopen.bmj.com/site/about/guidelines.xhtml

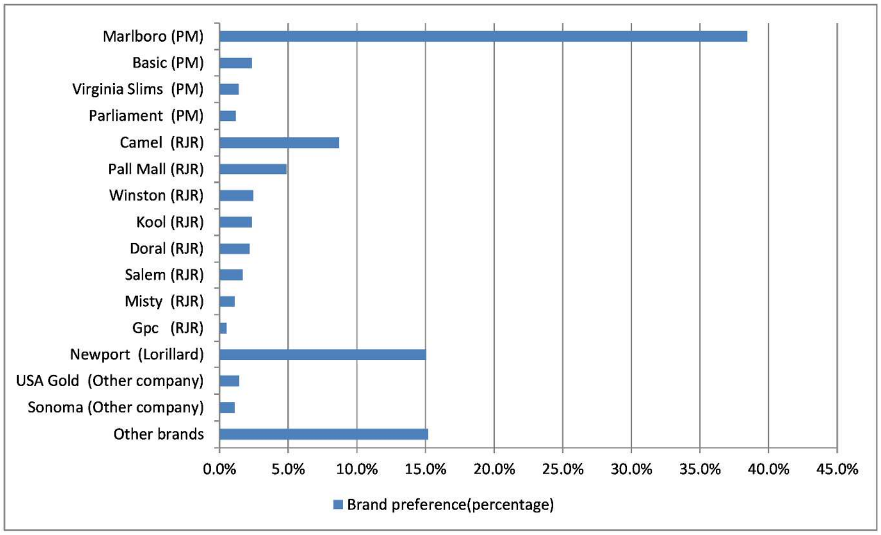

279x215mm (300 x 300 DPI)

For peer review only
